# BRD2 Compartmentalizes the Accessible Genome

**DOI:** 10.1038/s41588-022-01044-9

**Published:** 2022-04-11

**Authors:** Liangqi Xie, Peng Dong, Yifeng Qi, Tsung-Han S. Hsieh, Brian P. English, SeolKyoung Jung, Xingqi Chen, Margherita De Marzio, Rafael Casellas, Howard Y. Chang, Bin Zhang, Robert Tjian, Zhe Liu

**Affiliations:** 1 Janelia Research Campus, Howard Hughes Medical Institute, Ashburn, VA 20147, USA.; 2 Department of Molecular and Cell Biology, Li Ka Shing Center for Biomedical and Health Sciences, CIRM Center of Excellence, University of California, Berkeley, CA 94720, USA.; 3 Howard Hughes Medical Institute, Berkeley, CA 94720, USA.; 4 Departments of Chemistry, Massachusetts Institute of Technology, 77 Massachusetts Ave, Cambridge, MA 02139-4307, USA.; 5 Center for Personal Dynamic Regulomes and Howard Hughes Medical Institute, Stanford University, Stanford, CA 94305, USA.; 7 Lymphocyte Nuclear Biology, NIAMS and Center of Cancer Research, NCI, NIH, Bethesda, MD 20892, USA.; 8 Department of Immunology, Genetics and Pathology, Uppsala University, 751 08 Uppsala, Sweden.

**Keywords:** Genome organization, accessible chromatin, 3D ATAC-PALM, loop extrusion, Cohesin, BRD2, BRD4, Oligopaints, single-molecule imaging

## Abstract

Mammalian chromosomes are organized into megabase-sized compartments that are further subdivided into topologically associated domains (TADs). While the formation of TADs is dependent on Cohesin, the mechanism behind compartmentalization remains enigmatic. Here, we show that the bromodomain and extraterminal (BET) family scaffold protein BRD2 promotes spatial mixing and compartmentalization of active chromatin after Cohesin loss. This activity is independent of transcription but requires BRD2 to recognize acetylated targets through its double bromodomain and interact with binding partners with its low complexity domain. Notably, genome compartmentalization mediated by BRD2 is antagonized on one hand by Cohesin and on the other by the BET homolog protein BRD4, both of which inhibit BRD2 binding to chromatin. Polymer simulation of our data supports a BRD2-Cohesin interplay model of nuclear topology, where genome compartmentalization results from a competition between loop extrusion and chromatin state-specific affinity interactions.

In the past decade, the rapid development of chromosome conformation capture (3C) assays has provided comprehensive measurements of chromatin folding and genome organization with rich sequence information ^1-5^. The general model emerging from such studies is that the mammalian genome is organized into distinct architectural scales including compartments ^6^, topologically associated domains (TADs) ^7-9^, and loop domains ^10^. Specifically, the checkerboard-like pattern of alternating contact enrichment and depletion in Hi-C maps reflects the spatial arrangement of active euchromatin (A compartments) and inactive heterochromatin (B compartments) ^6,11^. However, the molecular basis underlying the formation of compartments, or compartmentalization, remains largely elusive. On the other hand, the formation of TAD and loop domain, evidently depends on architectural proteins including Cohesin and CTCF that likely function through the proposed loop extrusion mechanism ^12-14^. Emerging studies that coupled genome-wide Hi-C measurements or super-resolution imaging with Cohesin/CTCF degradation have satisfactorily validated several key predictions of the loop extrusion model. Specifically, Cohesin loss eliminates loop domains, reduces intra-TAD contacts and inter-TADs intermingling, underscoring its seminal role in genome organization ^15-21^. Moreover, CTCF loss abolishes chromatin interactions between CTCF motifs and reduces inter-TADs insulation ^16,21-24^.

One perplexing and converging observation from previous studies is that Cohesin loss promotes stronger checkerboard-like pattern in the Hi-C map, enhances chromatin compartmentalization, and causes enhancers from the same and different chromosomes to co-localize ^15,17^. Based on these results, it was proposed that genome compartmentalization is independent of Cohesin and presumably in competition with loop/TAD formation ^17^. Similarly, super-resolution imaging experiments have found that globular ‘TAD-like’ domain structures, nanodomains within TADs and chromatin domain interactions persist after Cohesin depletion ^21,25,26^, suggesting a Cohesin-independent mechanism for establishing such contacts. Polymer simulations revealed that chromatin is folded at various length scales via distinct mechanisms and that compartment-specific affinity interactions can operate in parallel with the loop extrusion to organize the genome ^27-30^. Recently, it was shown that the affinity interaction between heterochromatic regions is required for compartmentalization in heterochromatin ^11,31,32^. Another study suggests that L1 and B1/Alu repeats could demarcate the genome compartments and that the L1-derived transcripts could promote heterochromatin formation with the role of B1 in regulating active compartments undefined ^33^. Therefore, the molecular identities and mechanisms underlying the compartmentalization of active chromatin remain unclear, representing a major knowledge gap in understanding the 3D genome folding and function.

To dissect the molecular basis underpinning compartmentalization of mammalian chromosomes, we harnessed our recently-developed 3D ATAC-PALM imaging platform that can reconstruct the 3D nanometer architecture of the accessible genome by precise localization of high-density photoactivatable probes inserted into active chromatin ^24^. As previously described, the accessible genome is organized into distinct accessible chromatin domains (ACDs) which spatially encompass individual ATAC-rich, active domains presumably associated with A compartments ^24,34^. We now show that Cohesin loss triggers spatial mixing of ACDs without altering the compaction or the 3D volume of individual domains. With chemical-genetic perturbations, super-resolution imaging and Micro-C genomic interaction mapping, we identify the BET family scaffold protein BRD2 with the ability to promote spatial mixing and maintain the compaction of active chromatin upon Cohesin loss. In addition, we find that the BET homolog protein BRD4 antagonizes the binding of BRD2 to chromatin and inhibits its role in genome organization. Finally, polymer simulation incorporating both loop extrusion and chromatin state-specific affinity interactions recapitulates our experimental observations. The convergence between microscopy, genomics and simulation suggests that the interplay between BRD2 and Cohesin supplies an important function to maintain the stereotypical spatial arrangement and balanced compartmentalization of the 3D genome in mammalian cells.

## Results

### Cohesin loss triggers spatial mixing of ACDs without affecting their compaction

**Extended Data Fig.1 ∣:**
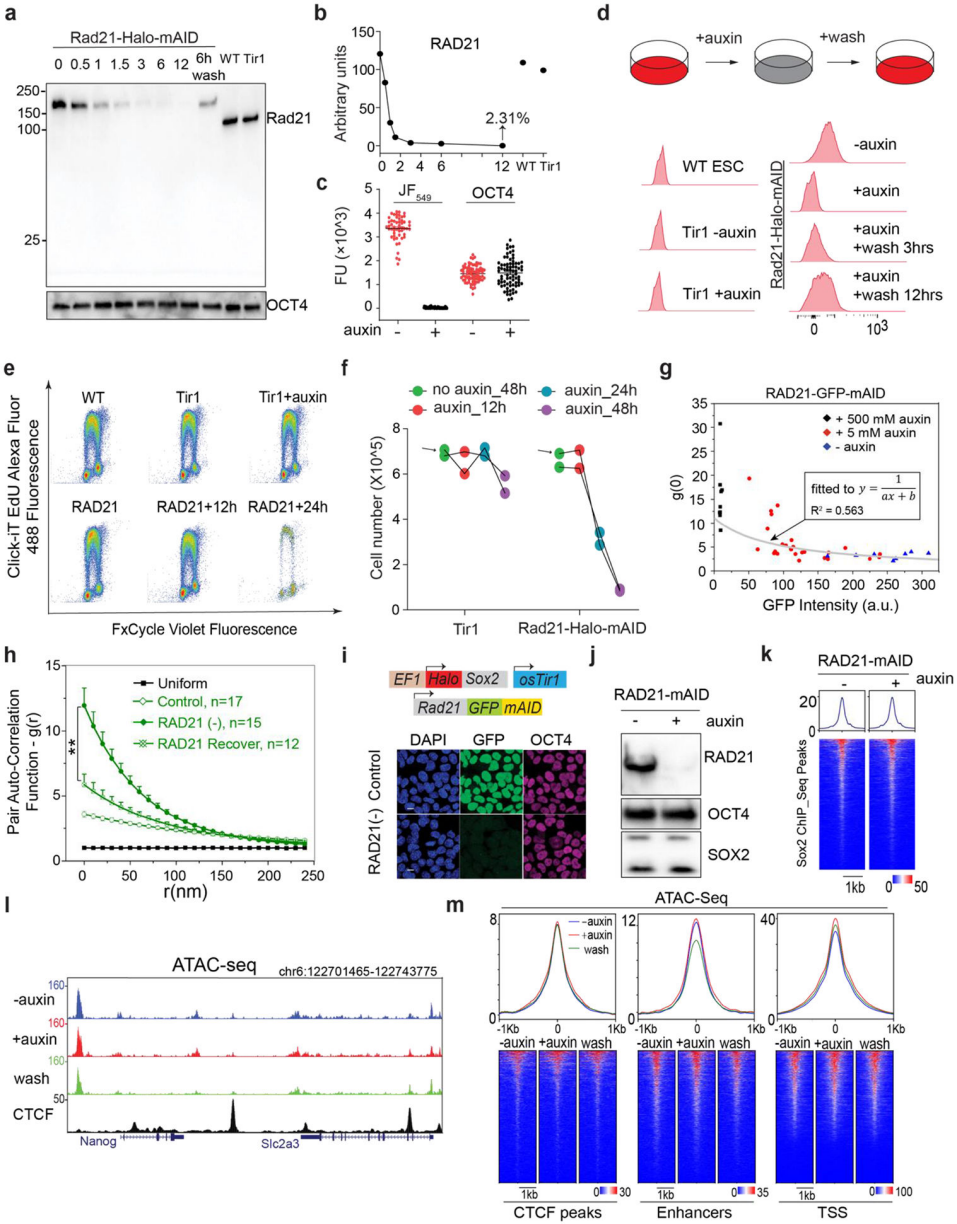
Efficient AID degron-mediated RAD21 depletion. Related to Fig.1. **(a)** Western blot (WB) analysis of protein levels of endogenous RAD21-HaloTag-mAID at indicated time points after the auxin treatment and wash off recovery. OCT4 protein was used as the internal control. **(b**) The WB intensity is represented as grey scale values for the corresponding WB bands in (**a**). **(c)** Single cell fluorescence intensity analysis of HaloTag-mAID-RAD21 and OCT4 protein levels after auxin treatment shown in Fig.1a. RAD21 was stained with the HaloTag ligand JF_549_. OCT4 was detected by immunofluorescence. FU, arbitrary fluorescent unit. The black line represents mean fluorescence value. **(d)** Flow cytometry analysis of RAD21 levels before (−auxin), after (+auxin) auxin treatment and during the recovery after auxin wash off. Cells were stained with 100nM HaloTag ligand JF_549_. The parent Tir1 ES cells (Tir1 − auxin; Tir1 + auxin) were used as the negative control (adopted from Extended data Fig.4d from our previous work ^24^). 50,000 gated live cells were recorded and analyzed for each condition. **(e)** DNA synthesis analysis of RAD21-HaloTag-mAID ESCs by the Click-iT EdU labeling kit at the indicated time points after auxin treatment. The same number (50,000) of cells were analyzed for all conditions. **(f)** Cell proliferation analysis of parental Tir1 (left) and RAD21-Halo-mAID (right) ESCs after auxin treatment. Cells were treated with auxin for indicated time points and the total cell number was measured after 48 hours. Data from two replicates were shown. **(g**) The dose-dependent effect of Cohesin depletion on global accessible chromatin clustering revealed by the inverse relationship between clustering amplitude (*A*) and residual RAD21 levels measured by RAD21-GFP-mAID fluorescence intensities (arbitrary fluorescent units). Specifically, two different auxin concentrations (5 μM and 500 μM) were used to generate a gradient of RAD21 level in single cells. **(h)** Pair auto-correlation function *g(r)* of ATAC-PALM localizations from RAD21-GFP-mAID ESCs under normal, auxin-treated and recovery conditions after auxin wash off (~24 hours). The error bars represent standard error (SE). The Mann-Whitney *U* test was performed. ** indicates p<0.01. **(i)** HaloTag-SOX2 fusion protein is stably expressed in the RAD21-mAID-GFP cells co-expressing the E3 ligase osTir1. Single cell fluorescence analysis of Cohesin levels upon acute auxin treatment. **(j**) WB analysis of RAD21, OCT4 or SOX2 upon auxin addition. SOX2 antibody detects both endogenous (lower band) and ectopic expressed HaloTag-SOX2 proteins (upper band). **(k)** Chromatin accessibility at SOX2 binding sites is not significantly affected by RAD21 depletion. The ATAC-seq enrichments around [−1kb 1kb] region centering SOX2 binding sites are color encoded and ranked by SOX2 ChIP-seq peak intensity. **(l**) ATAC-seq enrichment at a representative genomic region under normal (−auxin), auxin-treated (+auxin) and recovery conditions for RAD21-HaloTag-mAID cells. CTCF ChIP-seq track is displayed below as a reference. **(m)** RAD21-HaloTag-mAID cells were processed for genome-wide ATAC-seq analysis under conditions including without auxin, auxin treatment and washout after auxin treatment. RAD21 depletion does not noticeably change the chromatin accessibility at enhancers, promoters and insulator region.

**Fig.1 ∣:**
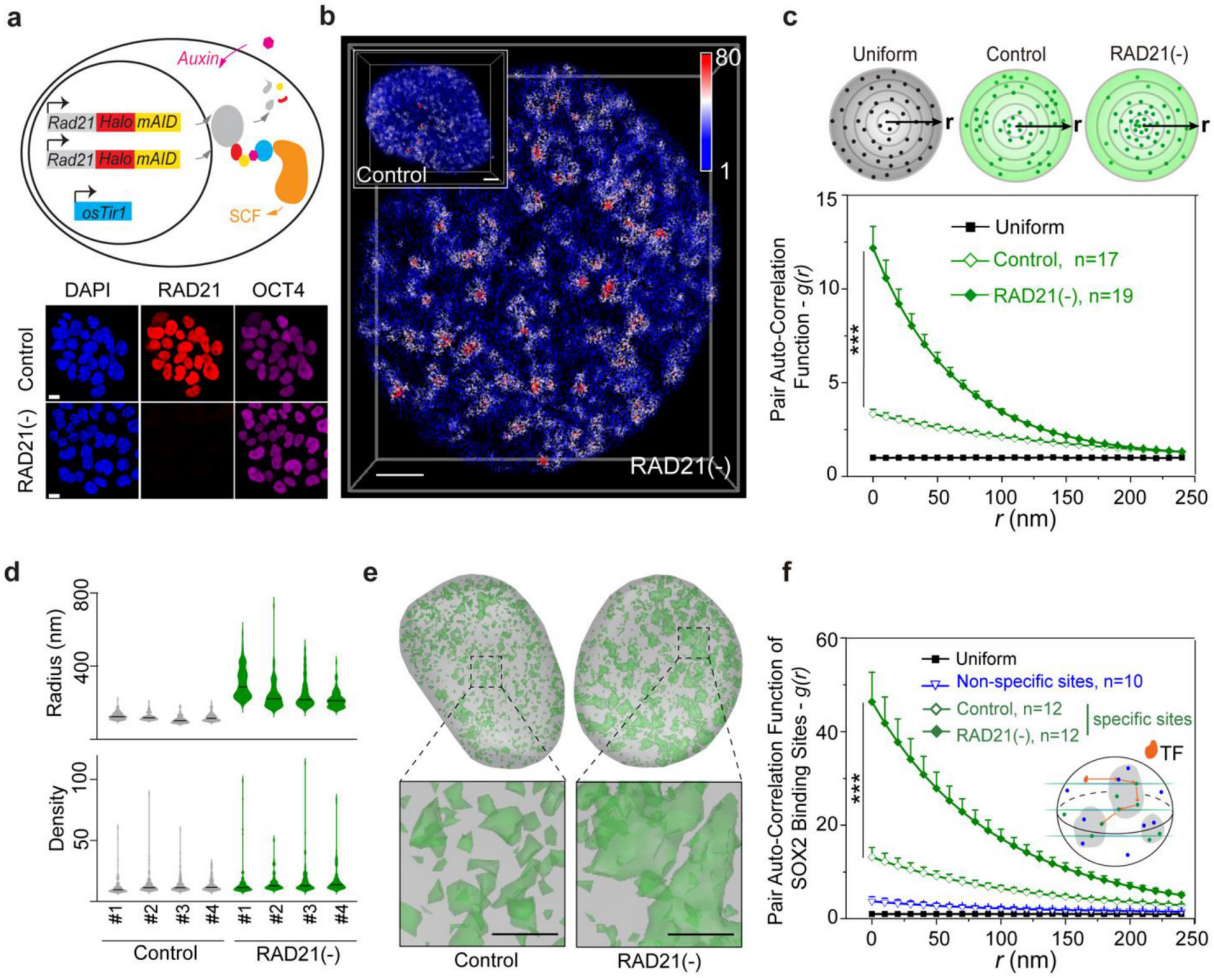
Cohesin prevents accessible chromatin from spatial mixing. **(a)** (Upper panel) A schematic of acute depletion of Cohesin in mouse ESCs by the auxin-induced protein depletion system. The mini auxin-inducible degron (mAID)-HaloTag was bi-allelically knocked into the C-terminus of *Rad21* gene (Cohesin subunit) by CRISPR/Cas9 in mESCs stably expressing the plant derived E3 ligase adaptor protein osTir1. (Lower panel) Adding plant derived hormone analogue (auxin) triggers rapid degradation of target protein RAD21 as revealed by single cell fluorescence imaging (HaloTag ligand JF_549_). OCT4 immunostaining was used as a control. Scale bar, 5 μm. **(b)** Single-cell illustration of 3D ATAC-PALM localizations upon RAD21 depletion. A wild type control cell is also shown in the upper left corner. The color bar indicates localization density calculated by using a canopy radius of 250 nm. The experiments have been independently repeated for four times. Also see the 3D rotatory presentation in Supplementary Movie 1. Scale bar, 2 μm. **(c)** RAD21 depletion promotes global increase of accessible chromatin clustering measured by pair auto-correlation function, which describes the similarity among observations. The top panel shows a simplified two-dimensional scheme for distribution of localizations in uniform (black dots, left) or wild type (green dots, middle) or RAD21 depleted (green dots, right) conditions. *g(r)* represents the pair autocorrelation function of distance *r* calculated from a given origin point inside the space. The error bar represents standard error (SE) of the mean and two-sided Mann-Whitney U test was applied for comparing data points at *g(0)*. **(d)** The violin plot of normalized radius (upper panel) or localization density (lower panel) of top 100 ranked ACDs among 4 individual cells for Control and RAD21 depleted conditions. The localization density was determined by the total number of ATAC-PALM localizations within a 250nm(radius) spherical region. The black bar indicates the median value for each data set. **(e)** 3D *iso*-surface reconstruction of ACDs (green) identified by using the DBSCAN algorithm for Control (left panel) and RAD21 depletion (right panel) conditions. The *iso*-surface in grey outlines the nuclear envelope. The lower panels show 4× magnification of local regions under each condition. Scale bar, 1 μm. **(f**) Enhanced clustering of transcription factor (TF) SOX2 stable binding sites upon RAD21 depletion. The pair auto-correlation function *g(r)* for stable SOX2 binding sites was calculated before and after RAD21 depletion. SOX2 binding events with dwell times longer than 4s were considered as stable binding events (see methods). The error bars represent standard error (SE) of the mean. The two-sided Mann-Whitney U test was used for statistical testing. The inset illustrates the 3D TF binding between specific (green) and non-specific (blue) binding sites.

To study how Cohesin organizes accessible chromatin, we first established a Cohesin-depletion system based on the auxin-inducible degron (AID) ^35^. Specifically, endogenous Cohesin subunit RAD21 was tagged with a HaloTag-miniAID (mAID) in a mouse embryonic stem cell (ESC) line stably expressing the rice F-box protein TIR1 (Fig.1a). The HaloTag-mAID tagging did not appear to influence the basal RAD21 protein levels (Extended Data Fig.1a-b) or cell proliferation without auxin treatment (Extended Data Fig.1e-f). Auxin treatment rapidly reduced RAD21 levels as shown by western blot, single cell fluorescence imaging and flow cytometry (Fig.1a, Extended Data Fig.1a-d). The depletion was reversible, as RAD21 protein levels quickly recovered after auxin washout (Extended Data Fig.1a, 1d). Acute loss of RAD21 did not cause noticeable changes in proliferation, cell cycle phasing or expression of pluripotency markers (Extended Data Fig.1e-f) whereas prolonged RAD21 loss (> 24 hours) did compromise proliferation and survival of ESCs (Extended Data Fig.1e-f).

3D ATAC-PALM imaging revealed that acute loss of Cohesin triggered prominent organizational changes in ACDs with markedly enhanced clustering of accessible chromatin (Fig.1b-c, Supplementary Movie 1). Importantly, the degree of clustering is inversely correlated with residual Cohesin levels in single cells, suggesting a dose-dependent effect (Extended Data Fig.1g). In addition, the increase in accessible chromatin clustering was reversed as RAD21 levels recovered after auxin washout (Extended Data Fig.1h), consistent with previous reports that loops and TADs rapidly re-establish after the recovery from Cohesin depletion ^15,36^.

Distinct from our previous observation that CTCF loss preferentially increased ATAC-PALM localization density in individual clusters ^24^, Cohesin loss selectively induced the formation of much larger and more connected clusters in the nucleus, as if multiple ACDs began to mix in space (Fig.1d-e). This is reminiscent of the formation of higher order enhancer hubs and enhanced compartmentalization of active chromatin after Cohesin loss ^15,17^. Because enhancers represent a subset of accessible chromatin, we next imaged the 3D SOX2 enhancer clusters in single live ESCs using a previously established lattice light-sheet based imaging strategy ^37^. We found that Cohesin removal induced spatial clustering of SOX2 stable binding sites (Fig.1f, Supplementary Movie 2) without perturbing SOX2 levels or its binding site accessibility (Extended Data Fig.1i-k), consistent with the enhanced clustering of accessible chromatin detected by 3D ATAC-PALM imaging. It is important to note that acute loss of Cohesin did not significantly affect chromatin accessibility at enhancers, promoters or insulators (Extended Data Fig.1l-m), suggesting that Cohesin removal profoundly altered the 3D spatial arrangement of *cis*-regulatory elements without affecting their accessibility in the linear genome.

**Extended Data Fig.2 ∣:**
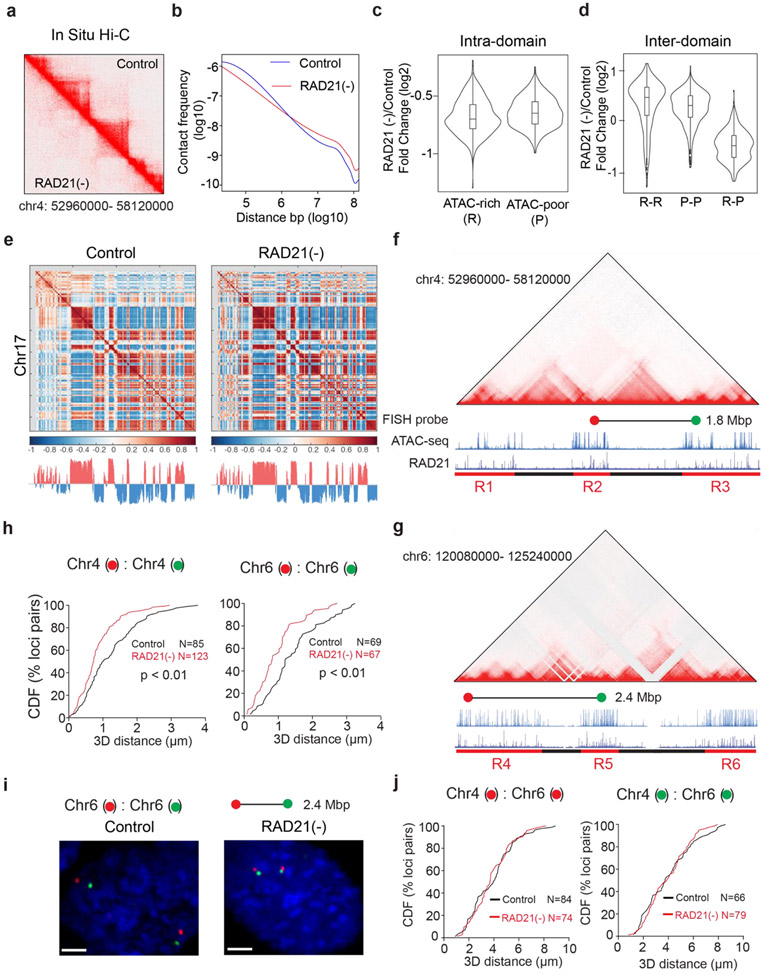
Validation of 3D ATAC-PALM observations by in situ Hi-C and OligoPaint FISH. Related to Fig.1. **(a)** Representative in situ Hi-C contact map from the chr4: 52960000- 58120000 genomic region. The upper triangle is control and lower triangle is after 6 hours RAD21 depletion. **(b)** Genome-wide contact probability decaying curve by in situ Hi-C. We found a lower contact frequency at a genomic distance smaller than ~1Mbp and higher contact frequency at distance larger than ~1Mbp after RAD21 depletion. The reduced contact frequency likely occurred in chromatin loops or TADs within compartments. **(c)** Violin plot of log2 scale fold change of total contacts from genome-wide intra segments (ATAC-rich (R) and ATAC-poor (P) based on chromatin accessibility from ATAC-seq data) from 6 hours RAD21 depletion compared to untreated control. **(d)** Violin plot of log2 scale fold change of total contacts from genome-wide adjacent inter segments (R-R, P-P, R-P) from RAD21 depletion vs untreated control. The contacts from neighboring R-R or P-P segments are increased after RAD21 depletion whereas those from adjacent R-P segments are decreased. **(e)** In situ Hi-C Pearson correlation matrix map of chromosome 17 for Control and RAD21 depletion for 6 hours. The compartmental scores of eigenvector PC1 value track was plotted on the bottom of each map. **(f-g)** Alignment of Hi-C heatmap, ATAC-seq, RAD21 ChIP-seq tracks for chromosome 4 **(f)** and chromosome 6 **(g).** DNA-FISH probes corresponding to loci pairs in the adjacent ATAC-rich segments are marked as red and green dots joined by a black line. The linear genomic distance between the two loci is indicated on the side. ATAC-rich (R1-6; red) segments are underlined. The chromatin length of each segment are R1(633kb), R2(643kb), R3(1120kb), R4(1250kb), R5(640kb), R6(990kb). **(h**) 3D physical distances between intra-chromosomal loci pairs are significantly reduced upon Cohesin depletion. Specifically, cumulative distribution function (CDF) of loci pair distances was plotted before and after Cohesin depletion. The two-sample Kolmogorov-Smirnov test was performed (p < 0.01). **(i)** Representative two-color 3D Oligopaint FISH images are shown for the loci pair in Chr6 under Control (left panel) and RAD21-depleted (right panel, auxin treatment) conditions. Scale bar, 2 μm. **(j)** The 3D physical distances between inter-chromosomal loci pairs are not significantly altered upon Cohesin depletion. CDF for the 3D distances of loci pairs between two chromosomes (red to red, green to green) were measured before (black) and after (red) RAD21 depletion. The number of measurements is indicated below the graph. The two-sample Kolmogorov-Smirnov test was performed (p>0.05).

To validate these structural changes observed by microscopy, we performed *in situ* Hi-C genomic experiments and found that Cohesin depletion eliminated chromatin loops and TADs as expected (Extended Data Fig.2a). Additionally, while short-range intra-compartment contact frequencies were reduced, long-range (>~1Mbp) contact frequencies between active compartments were increased upon Cohesin loss (Extended Data Fig.2b-d) consistent with our imaging and previous Hi-C observations ^15-17^. To further validate this conclusion, we measured the 3D distance between loci pairs in neighboring active domains with Oligopaint DNA FISH (Extended Data Fig.2f-g) and found that Cohesin loss reduced physical distances between active domains *in cis* (from the same chromosome) but not *in trans (*from different chromosomes) (Extended Data Fig.2h-j). Together, these single-cell microscopy measurements showed high degree of consistency with cell-population based Hi-C genomic results, demonstrating the crucial role of Cohesin in maintaining the physical separation between active domains in the genome.

**Extended Data Fig.3 ∣:**
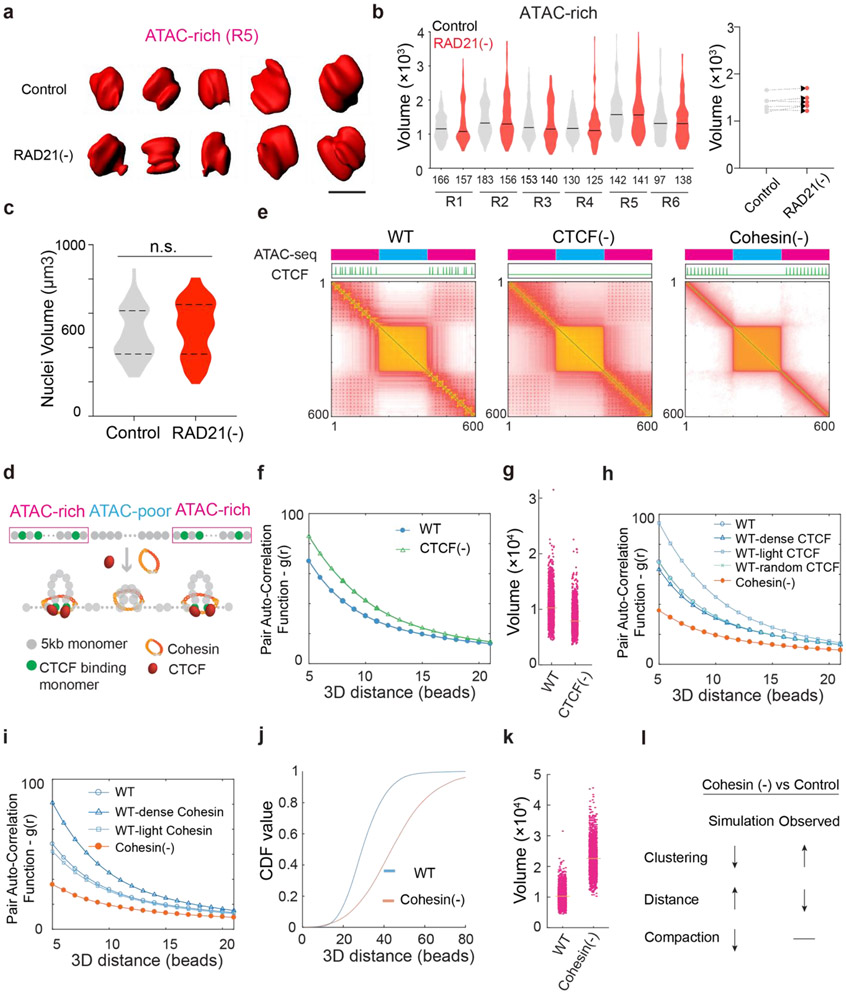
Polymer model simulation based on loop extrusion only. Related to Fig.1. **(a)** Representative *iso*-surfaces of an ATAC-rich segment (R5, red) in Control and RAD21 depletion conditions. Scale bar, 1 μm. **(b)** (Left)Violin plot of 3D volumes (the number of voxels) of six ATAC-rich segments (R1-R6) before and after RAD21 depletion. The number of alleles analyzed is indicated at the bottom. The black bar indicates the median value for each data set. The Mann-Whitney U test was applied, and we do not find statistical difference for the 3D volume distribution before and after RAD21 depletion. (Right)The paired mean value of 3D volume (number of voxels) for the 6 ATAC-rich regions before and after RAD21 depletion is plotted. **(c)** Quantification of nuclei volume as determined by DAPI signal before (n=65) and after (n=69) RAD21 depletion for 6 hours. Dashed lines indicate the first and third quartiles. No statistical significance was found (n.s., not significant with p value 0.21) by the non-parametric Mann-Whitney test. **(d)** The schematic overview of the polymer simulation. The chromatin polymer is modeled as beads on a string. We simulated two ATAC-rich segments (pink) on both ends and one ATAC-poor segments (blue) in the middle. Each segment is simulated with 200 monomers (grey) each representing a 5kb genomic segment. In each ATAC-rich segment, every 15th bead was assigned as CTCF binding sites (CTCF monomer, green) to insulate the loop extrusion by Cohesin. See details of simulation parameter set up in the Methods. **(e**) Contact probability map for the wild type (WT), CTCF depletion and Cohesin depletion conditions by considering loop extrusion mechanism alone. Two panels shown on the top are distinct ATAC segments and the relative positions of CTCF binding beads in the polymer model. **(f-g)** Distributions for the volume of the convex hull (**f**) or the pair auto-correlation function *g*(*r*) (**g**) of the ATAC-rich segments after CTCF depletion base on the ‘loop-extrusion only’ simulation model. **(h-i)** Simulation results are robust with respect to the setup of the model. (**h**) *g(r)* for varying the location and density of CTCF-binding sites for the wild type (blue) compared to the Cohesin depletion (orange). Dense, normal, light CTCF densities correspond to 36, 24 and 12 CTCF sites on the chromatin, respectively. Random CTCF sites correspond to randomly generate 24 CTCF sites on the chromatin. (**i**) Pair auto-correlation function *g(r)* for varying the number of Cohesin molecules on the chromatin for the WT ( blue) compared to the Cohesin depletion (orange). Dense, normal, light Cohesin densities correspond to the extrusion separation of 45kb, 90kb and 180kb, respectively. **(j-k)** The 3D loci pair distance from neighboring ATAC-rich segments **(j)** and distributions for the volume of the convex hull of ATAC-rich segments **(k)** were extracted in WT and Cohesin depleted conditions in which the ‘loop-extrusion only’ simulation model is considered. The average volume (yellow bar) for ATAC-rich segments of the Cohesin depletion is larger than that of the WT condition. **(l)** Summary of accessible chromatin clustering, neighboring distance, and compaction based on ‘loop-extrusion only’ polymer model simulation compared with experimental observations.

Previously, we demonstrated with Oligopaints that CTCF loss compacts active domains (~sub megabase) ^24^. To examine whether Cohesin influences chromatin compaction, we utilized the same strategy to measure the volumetric changes of 6 active domains upon Cohesin loss. Loop extrusion by Cohesin could promote long-range chromatin interactions ^13,14,27^. When this function is blocked, chromatin is predicted to undergo decompaction ^18,38^. Surprisingly, we found that Cohesin removal did not significantly alter compaction of tested active domains (Extended Data Fig.3a-b) nor the size of the nucleus (Extended Data Fig.3c), in agreement with previous reports ^20,26,39^ and ruling out the possibility that the enhanced clustering of accessible chromatin is caused by changes in overall chromatin density in the nucleus.

To probe physical mechanisms underlying these observations, we next performed loop-extrusion simulation on polymer fragments consisting of alternating active and inactive segments (Extended Data Fig.3d-e). The loop extrusion model was able to recapitulate the impact of CTCF loss that we observed ^24^, including the increase in accessible chromatin clustering and the reduction in volumes of active domains (Extended Data Fig.3f-g). However, the simulation was unable to recapitulate our experimental observations after Cohesin loss. Specifically, the loop extrusion model predicted a decrease in accessible chromatin clustering, increased distances between active segments, and chromatin decompaction (Extended Data Fig.3h-l). These apparent discrepancies between theory and experimental data reinforced the notion that Cohesin-independent mechanism(s) must be at play to regulate the organization of the accessible genome.

### Spatial mixing of ACDs is independent of transcription but involves BET family proteins

**Extended Data Fig.4 ∣:**
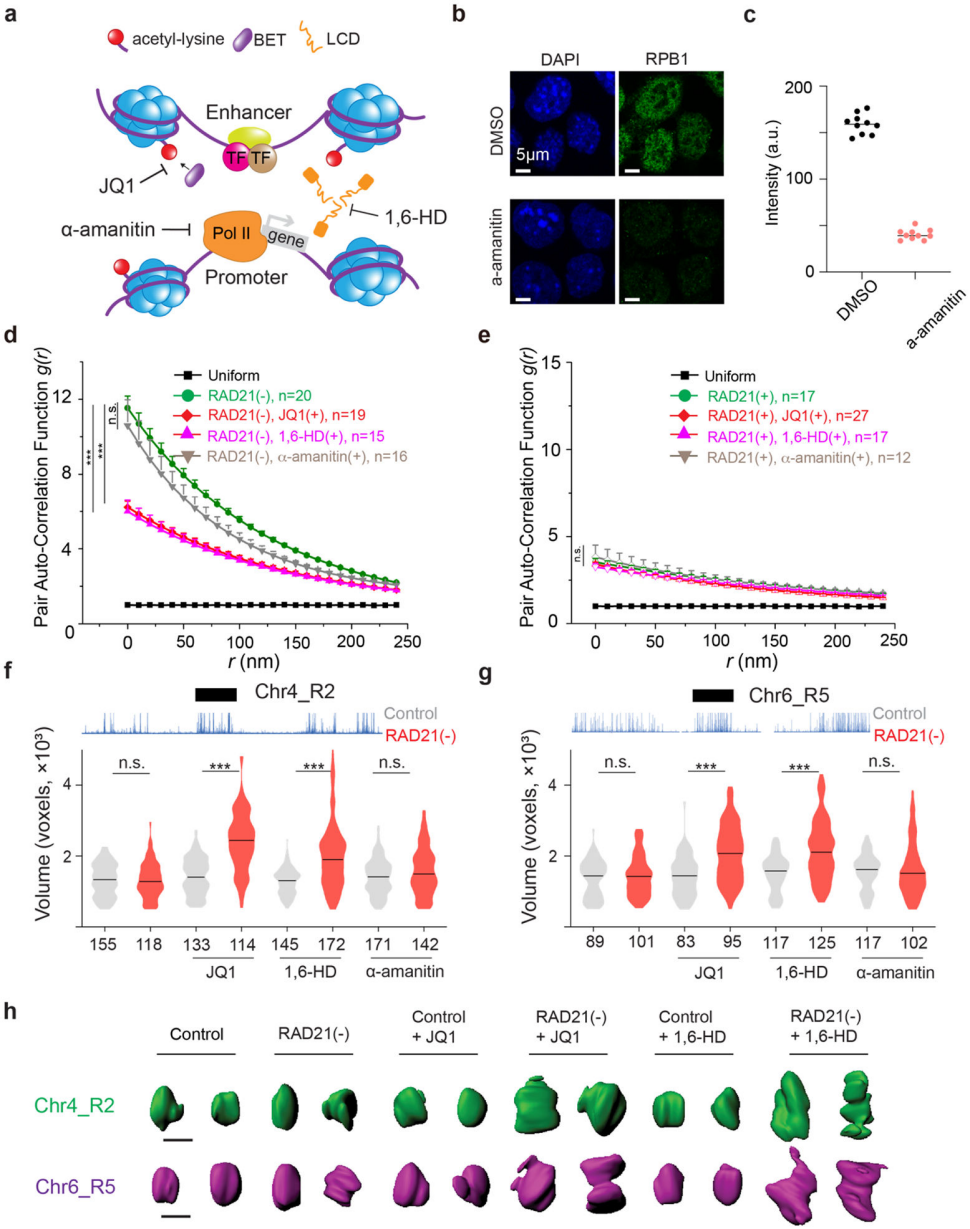
Regulation of accessible chromatin domains involves BET family proteins. Related to Fig.2. **(a)** A schematic of multiple small molecule inhibitors to target highly enriched nuclear proteins in the enhancer and promoter regions within ACDs. Alpha-amanitin inhibits the RNA Polymerase II by selectively targeting its largest subunit Rpb1 for degradation. JQ1 specifically blocks BET proteins from binding to acetyl-lysine residuals on target proteins (e.g., nucleosomes). 1,6-hexanediol (1,6-HD) targets the protein-protein interactions (e.g., the Mediator complex) often mediated by low complexity domains (LCDs). **(b-c)** Immunofluorescence analysis of Rbp1 before and after 5 hours of alpha-amanitin treatment. **(b)** Cells were treated with alpha-amanitin at a final concentration of 100 μg/ml for 5 hours and then fixed for immunofluorescence analysis by using purified anti-RNA Polymerase II RPB1 antibody (H14, BioLegend) with a dilution of 1:500. Scale bar, 5μm. The experiments have been independently repeated for three times. **(c)** Quantification of fluorescence intensity values over 10 individual cells for each condition. **(d)** Disrupting BET-chromatin interactions or LCD mediated protein-protein interactions by JQ1 or 1,6HD, respectively, reduces accessible chromatin clustering measured by 3D ATAC-PALM after Cohesin depletion whereas alpha-amanitin treatment had no significant effect. *g(r)* curves were plotted for indicated conditions. The non-parametric two-sided Mann-Whitney U test was used for statistical testing. **(e)** Treatment of JQ1, 1,6HD or alpha-amanitin does not reduces accessible chromatin clustering in the presence of Cohesin. *g(r)* curves were plotted for indicated conditions. The two-sided Mann-Whitney U test was used for statistical testing. **(f-g)** Violin plot of 3D volumes (the number of voxels) of two ATAC-rich segments R2 (**f**) and R5 (**g**) before (grey) and after (red) auxin-mediated RAD21 depletion with or without JQ1(1μM, 12 hours), 1,6-HD (2%, ,1.5 hours) or alpha-amanitin (100μg/mL, 5 hours) treatment. The number of alleles analyzed is indicated at the bottom. The black bar indicates the median value for each data set and Mann-Whitney U test was applied for statistical test. ***, p < 0.001; n.s., not significant. **(h**) Representative *iso*-surfaces of an ATAC-rich segments R2 (green) and R5 (magenta) in control, JQ1 (1μM, 12 hours), and 1,6-HD (2%, ,1.5 hours) treated cells before and after auxin-mediated RAD21 depletion. Scale bar, 1 μm.

The transcriptional state has been found as a predictor of compartmentalization in several eukaryotic models ^5,40^. As acute loss of Cohesin does not significantly affect levels of global gene expression ^15^, we next tested whether transcriptional activities could be responsible for spatial mixing of ACDs upon Cohesin loss. We found that treatment with RNA Pol II inhibitor alpha-amanitin ^41,42^ and the subsequent degradation of Rbp1 (Extended Data Fig.4a-c) affected neither the clustering nor the compaction of accessible chromatin after Cohesin removal, suggesting a transcription-independent mechanism (Fig.4d-e). Our observation is consistent with recent high-resolution chromatin interaction studies showing that transcriptional inhibition had negligible impacts on the formation of compartments ^43,44^.

**Extended Data Fig.5 ∣:**
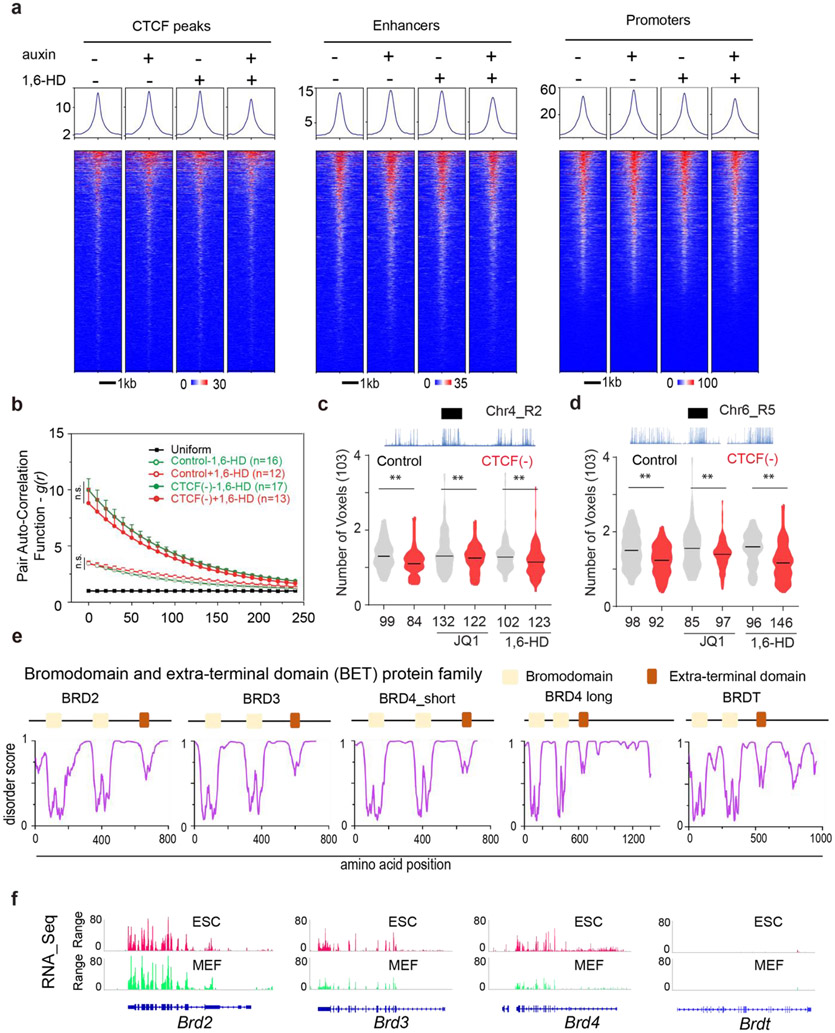
BET family proteins shape ACD organization in the absence of Cohesin. Related to Fig.2. **(a)** Low-dose 1,6 Hexanediol (1,6-HD,2%) treatment for 1.5 hours with or without Cohesin depletion did not significantly affect chromatin accessibility in the linear genome. The heatmaps of ATAC-seq enrichment at CTCF sites, enhancers and promoters are shown for WT, Cohesin depletion, 1,6 HD treatment, or a combination of both. **(b)** Disrupting BET-chromatin interactions by JQ1 had no significant impact on accessible chromatin clustering after CTCF depletion. *g(r)* curves were plotted for indicated conditions. Specifically, 1μM JQ1 treatment for 12 hours was applied to both control and CTCF depleted cells (6-hour depletion). The two-sided Mann-Whitney U test was used for statistical testing. **(c-d)** Violin plot of 3D volumes (the number of voxels) of two ATAC-rich segments R2 (**c**) and R5 (**d**) before (grey) and after (red) auxin-mediated CTCF depletion with or without JQ1 or 1,6HD treatment. The number of alleles analyzed is indicated at the bottom. The black bar indicates the median value for each data set and Mann-Whitney U test was performed for the statistical analysis. **(e**) The disordered region predicted from PONDR program (Predictor of Natural Disordered Regions) for BET family proteins using the VSL2 algorithms. Except for the double bromodomain and the extra-terminal domain, other regions of BET proteins are predicted to be highly disordered with high disorder score. **(f**) RNA-seq analysis of the expression level of BET family genes in mouse ESCs and mouse embryonic fibroblasts (MEF). Except for the *Brdt* gene, *Brd2/3/4* are all actively transcribed in ESCs. RNA-seq data was derived from GSM723776 and GSM723775.

A surge of recent studies reported that diverse nuclear localized proteins (transcription factors, cofactors and histone modification readers) assemble into dynamic clusters or hubs, via weak, multivalent interactions ^45-48^. To test whether multivalent interactions are involved, we chemically perturbed cells with small molecule inhibitors that disrupt protein-protein and protein-chromatin interactions monitored by 3D ATAC-PALM and Oligopaint FISH imaging. We identified two chemicals - aliphatic alcohol 1,6-hexanediol (1,6-HD) and JQ1 that selectively reduced the spatial mixing of ACDs and induced their decompaction in Cohesin-depleted but not in control cells (Extended Data Fig.4d-h, Extended Data Fig.5a-c). The effect of 1,6-HD treatment was somewhat predictable, as it nonspecifically disrupts hydrophobic protein interactions often mediated by low complexity domains (LCDs) ^49^. Nonetheless, this result confirmed our view that protein-protein interactions must be involved in the spatial mixing of ACDs after Cohesin loss. The observed effects of JQ1 became of particular interest, because it selectively inhibits the binding of a small set of BET family proteins (BRD2, BRD3, BRD4 and BRDT) to acetylated targets - mainly acetylated lysine residues on histone tails associated with active chromatin ^50^. These BET family proteins are predicted to contain large LCD segments with high degree of disorder (Extended Data Fig.5e) and are important transcriptional co-regulators with indispensable and non-overlapping functions in development and human diseases such as obesity, inflammation and cancer ^51^. We subsequently focused on BRD2, BRD3 and BRD4 as only these three are expressed in mouse ESCs (Extended Data Fig.5f).

### BRD2 maintains the compaction of ACDs and promotes their interactions in the absence of Cohesin

**Extended Data Fig.6 ∣:**
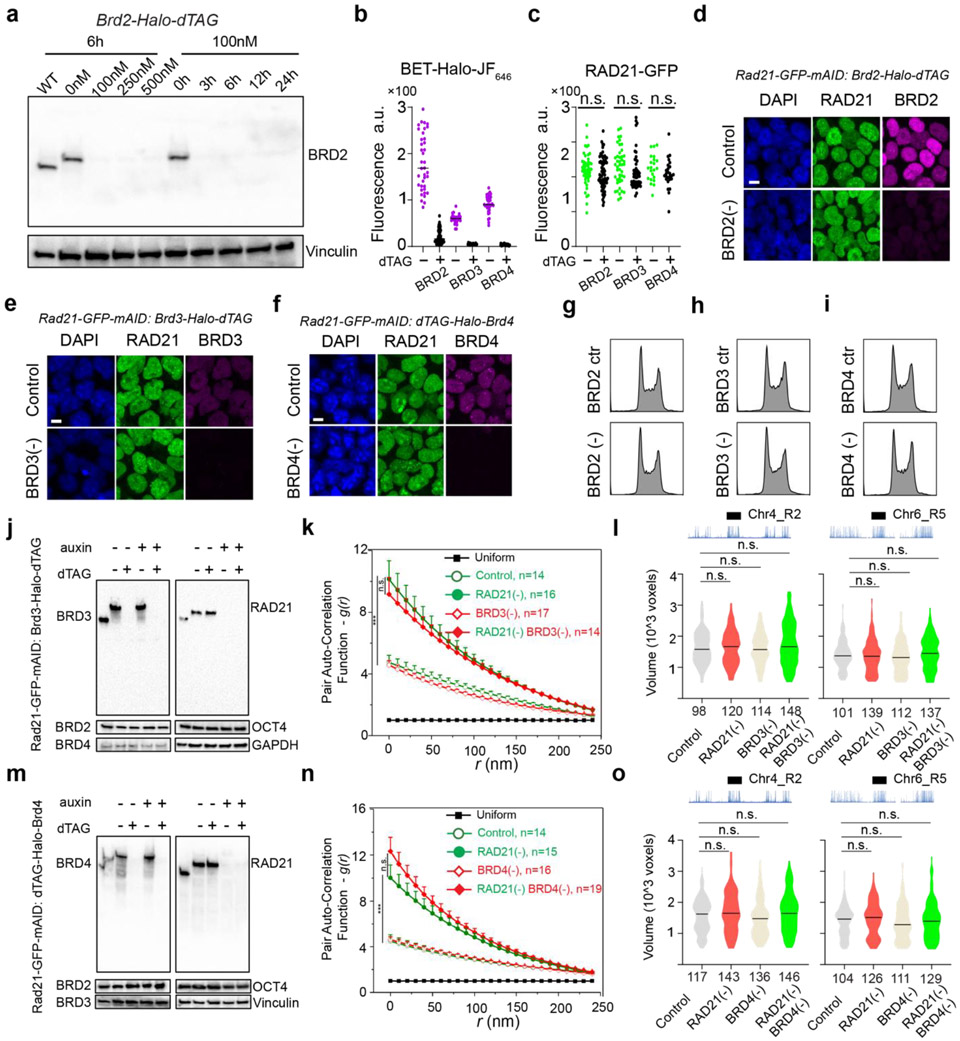
Characterization of acute BET family protein depletion on the spatial organization of the accessible genome. Related to Fig.2. **(a**) WB test of time and dose-dependent degradation of protein levels of endogenous BRD2-dTAG. 100nM dTAG13 is sufficient to deplete BRD2 as early as 3 hours. We used 100nM dTAG13 treatment for 6 hours throughout the current study. **(b-c**) Quantification of single cell fluorescence of endogenously labeled HaloTag-BRD2/3/4-using HaloTag ligand JF_646_ before and after dTAG13 treatment for 6 hours **(b).** RAD21 GFP signal serves as the control **(c).** **(d-f**) Representative images of acute degradation of BRD2 (**d**), BRD3 (**e**) and BRD4 (**f**) after 6 hours of dTAG13 treatment. Endogenous BET proteins were engineered with a HaloTag and labeled with 500nM JF_646_. The RAD21 level was monitored by GFP. Scale bar,5 μm. **(g-i)** Cell cycle analysis after 6 hours depletion of BRD2, BRD3 or BRD4 by propidium iodide staining. Histograms of the DNA content distribution measured by propidium iodide staining are shown. **(j**) WB analysis of protein levels of endogenous BRD3-dTAG or RAD21-AID individually or in combination after 6 hours of dTAG13 or auxin treatment, respectively. **(k**) Depletion of BRD3 for 6 hours did not significantly reduce the accessible chromatin clustering after RAD21 depletion. *g(r)* curves were plotted for indicated conditions. The two-sided Mann-Whitney U test was used for statistical testing. **(l)** Violin plot of 3D volumes (the number of voxels) of two ATAC-rich segments R2 (left) and R5 (right) before (grey) and after (red) BRD3 depletion, RAD21 depletion or in combination. The number of alleles analyzed is indicated at the bottom. The black bar indicates the median value for each data set and Mann-Whitney U test was performed for the statistical analysis. n.s.,not significant. **(m)** WB analysis of protein levels of endogenous BRD4-dTAG or RAD21-AID individually or in combination after 6 hours of dTAG13 or auxin treatment, respectively. **(n**) Degradation of BRD4 for 6 hours did not significantly reduce but instead had a trend to increase the accessible chromatin clustering after RAD21 depletion. *g(r)* curves were plotted for indicated conditions. The two-sided Mann-Whitney U test was used for statistical testing. **(o)** Violin plot of 3D volumes (the number of voxels) of two ATAC-rich segments R2 (left) and R5 (right) before (grey) and after (red) BRD4 depletion, RAD21 depletion or in combination. The number of alleles analyzed is indicated at the bottom. The black bar indicates the median value for each data set and Mann-Whitney U test was performed for the statistical analysis.

**Fig.2 ∣:**
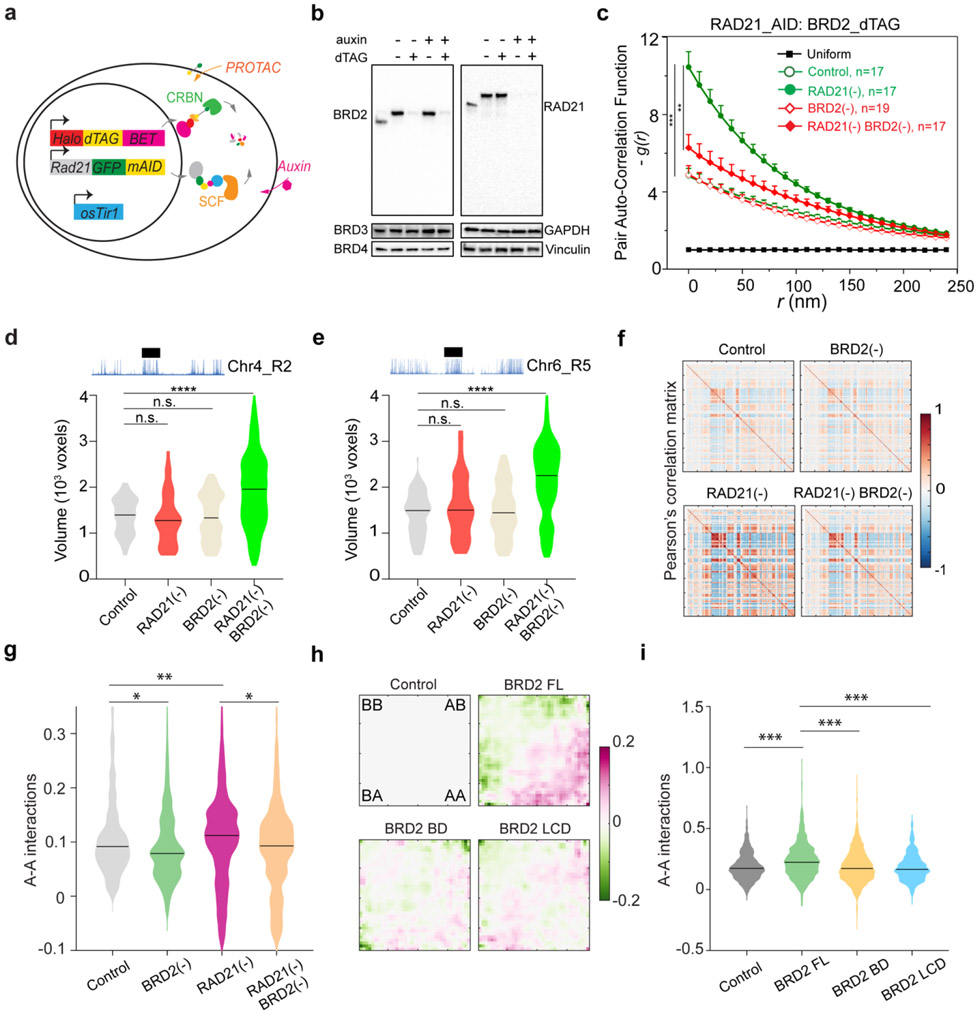
BRD2 mixes and compacts active compartments in the absence of Cohesin. **(a)** A schematic of the dual targeted protein degradation strategy. In *Rad21-eGFP-mAID* cells, a FKBP^F36V^ based degron (dTAG) linked to a HaloTag was bi-allelically knocked into endogenous BET family genes (*Brd2, Brd3 and Brd4*) by the CRISPR/Cas9 genome editing method (only one engineered allele is shown). Adding cell membrane permeable dTAG13 ligand into the culture will bring the dTAG labeled BET proteins into close proximity to the Cereblon (CRBN) E3 ligase for proteasome-mediated protein degradation orthogonal to the mAID system. **(b)** Western blot (WB) analysis of protein levels of endogenous BRD2-dTAG or RAD21-AID individually or in combination after 6 hours of dTAG13 or auxin treatment, respectively. Rapid depletion of BRD2 or RAD21 individually or together does not impact the protein level of other BET proteins (BRD3 or BRD4). **(c)** Degradation of BRD2 for 6 hours significantly reduced the accessible chromatin clustering after RAD21 depletion. *g(r)* curves were plotted for indicated conditions. **(d-e)** Violin plot of 3D volumes (the number of voxels) of two ATAC-rich segments R2 (**d**) and R5 (**e**) before and after BRD2 depletion, RAD21 depletion or in combination. The black bar indicates the median value for each data set and Mann-Whitney U test was performed for the statistical analysis. The number of analyzed alleles for the ATAC-rich segment (Chr4-R2) are : Control (n=93), RAD21 depletion (n=129), BRD2 depletion (n=116), or dual depletion of both RAD21 and BRD2 (n=119). The number of analyzed alleles for the ATAC-rich segment (Chr4-R5) are : Control (n=91), RAD21 depletion (n= 85), BRD2 depletion (n=99), or dual depletion of both RAD21 and BRD2 (n=121). **(f)** Pearson’s correlation matrix of the whole chromosome 17 for BRD2 or RAD21 depletion alone or in combination for 6 hours from Micro-C experiments. Dual BRD2 and RAD21 depletion reduced the enhanced compartmentalization after Cohesin depletion alone. **(g**) Quantification of the digitalized A-A compartmental interactions (log10 value of observed/expected) after BRD2 or RAD21 depletion alone or in combination from Micro-C experiments. The black solid line in each violin plot represents the median value. **(h-i)** Differential saddle plot analysis **(h)** and quantitative A-A compartmental interactions (log10 value of observed/expected) analysis **(i)** by Micro-C for cells stably expressing empty vector, full length (FL), N-terminal double bromodomain (BD) and C-terminal low complexity domain (LCD) of BRD2. The non-parametric two-sided Mann-Whitney U test was used for statistical testing. *, p < 0.05; **, p < 0.01; ***, p < 0.001; ****, p < 0.0001.

To identify which BET scaffold protein(s) might be involved in organizing accessible chromatin, we harnessed an orthogonal targeted protein degradation system - proteolysis targeting chimeras (PROTACs)^52^ to deplete individual BET family member, in parallel with the auxin-induced Cohesin depletion system (Fig.2a). We observed rapid and specific depletion of BRD2/3/4 proteins comparable to auxin-triggered Cohesin degradation (Fig.2b; Extended Data Fig.6a-f, Fig.6j, Fig.6m). Acute BET protein depletion up to 6 hours did not noticeably change the cell cycle progression (Extended Data Fig.6g-i). We initially hypothesized that BRD4 would most likely be involved, owing to its archetypical LCD that can form phase-separated droplets *in vitro* and puncta *in vivo* to promote chromatin interactions ^11,27,47^. To our surprise, BRD4 depletion did not reduce the clustering of accessible chromatin in Cohesin-depleted cells (Extended Data Fig.6n). In contrast, acute loss of BRD2 reversed spatial mixing of ACDs after Cohesin loss (Fig.2c), while BRD3 degradation had no significant effect (Extended Data Fig.6k). In agreement with the specific role of BRD2 in accessible chromatin clustering, we also found that depletion of BRD2 but not that of BRD4 or BRD3 led to decompaction of active domains upon Cohesin loss (Fig.2d-e; Extended Data Fig.6l, Fig.6o).

**Extended Data Fig.7 ∣:**
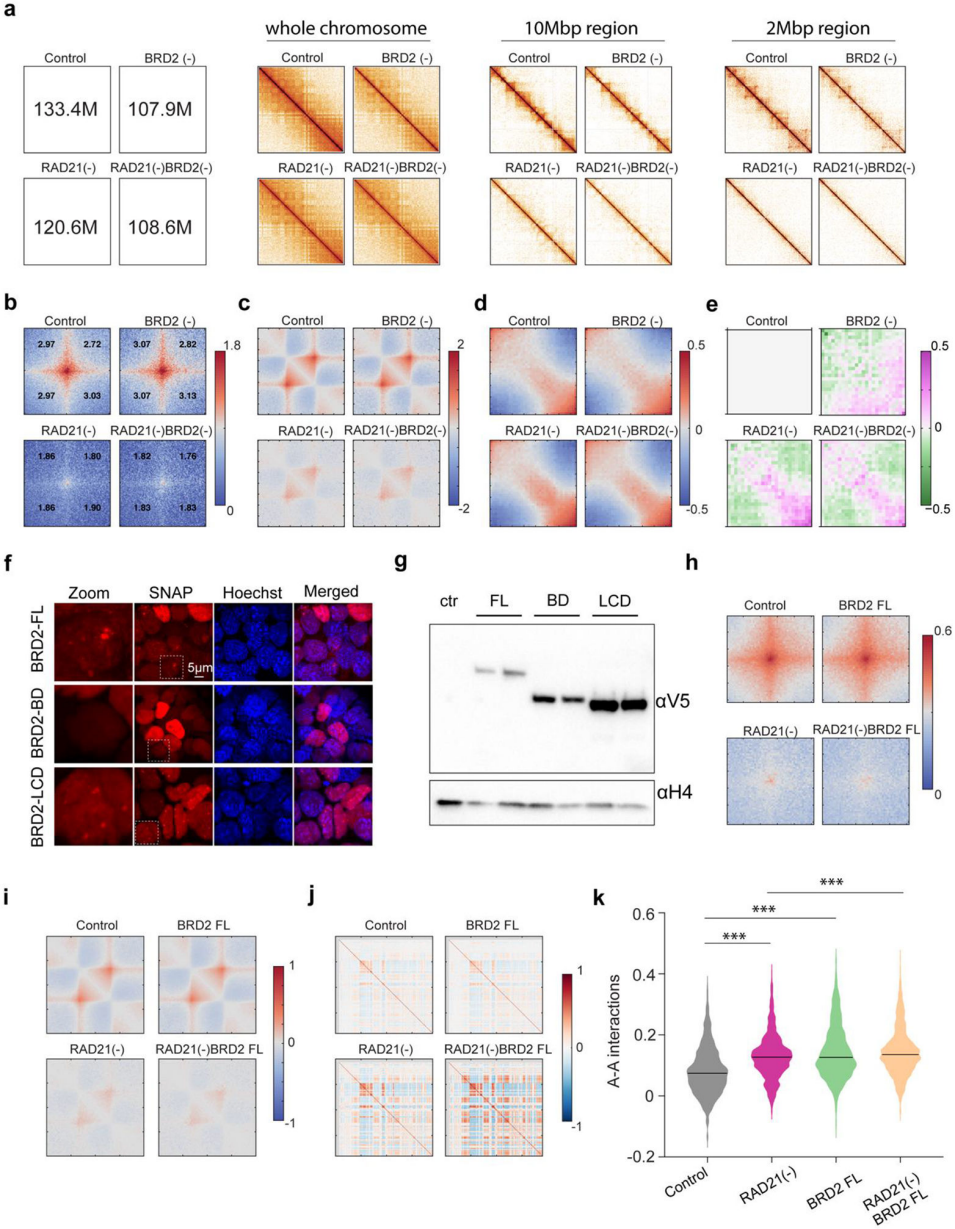
The BET family protein BRD2 contributes to chromatin compartmentalization. Related to Fig.2. **(a**) Snapshots of Micro-C contact maps at different size scales (whole chromosome, 10Mbp, 2Mbp) for Control, Cohesin depletion, BRD2 depletion or in their combination. The number of reads for each condition analyzed are indicated inside the box region in the left panel (M, million reads). Whole chromosome, chromosome 3; 10-Mb region, chromosome1:184M-194M; 2-Mb region, chromosome1:119M-121M. **(b-d**) Micro-C analysis of loops (**b**), TADs **(c)** and compartments (**d**) after Cohesin depletion, BRD2 depletion or in their combination. See more details in the Micro-C analysis in the methods section. **(e)** Differential saddle plot analysis of Cohesin depletion, BRD2 depletion or in their combination compared to non-treated control cells. **(f)** Live cell analysis of cells stably expressing the V5-SNAP tagged full length (FL), N-terminus double bromodomain (BD) and the C-terminus low complexity domain (LCD) of mouse BRD2. Cells were stained with SNAP JF_552_ ligand and counterstained with Hoechst 33342. First column indicates the zoomed-in view of cells marked by the while box in the second column. **(g)** WB analysis of cells from **(f)** analyzed by ant-V5 antibody and the anti-histone H4 antibody. **(h-i**) Micro-C analysis of loops (**h**) and TADs **(i)** for cells stably expressing FL BRD2 with or without Cohesin depletion. Ectopic expression of FL BRD2 does not restore the loss of Cohesin-dependent chromatin loops or architectural domains. **(j**) Micro-C analysis of compartments by the Pearson correlation matrix (chromosome 17) for cells stably expressing FL BRD2 with or without Cohesin depletion. **(k)** Quantification of the digitalized A-A compartmental interactions (log10 value of observed/expected) after ectopically expressing FL BRD2 with or without RAD21 depletion from Micro-C experiments. The black solid line in each violin plot represents the median value. *** indicates p < 0.001 by the non-parametric Mann-Whitney test.

To independently validate our imaging observations, we performed Micro-C experiments with BRD2 and Cohesin subunit-RAD21 acutely degraded individually or together. Micro-C showed that Cohesin loss eliminated chromatin loops/TADs and promoted interactions between active compartments as previously described (Fig.2f, Extended Data Fig.7a-e)^15-17^. Importantly, we found that simultaneous BRD2 and Cohesin depletion weakened the enhanced checkerboard pattern and reduced interactions between active compartments, in agreement with our imaging data (Fig.2f-g). In addition, ectopic expression of full length BRD2 but not the mutants lacking the double bromodomain or LCD was able to promote the long-range compartmental interactions (Fig.2h-l, Extended Data Fig.7f-k), suggesting that both BRD2-chromatin and BRD2-protein interactions are involved. These converging results from microscopy and genomic experiments reinforce the notion that BRD2 mixes and compacts active chromatin in the absence of Cohesin.

### The interplay between BRD2 and Cohesin regulates genome topology

**Extended Data Fig.8 ∣:**
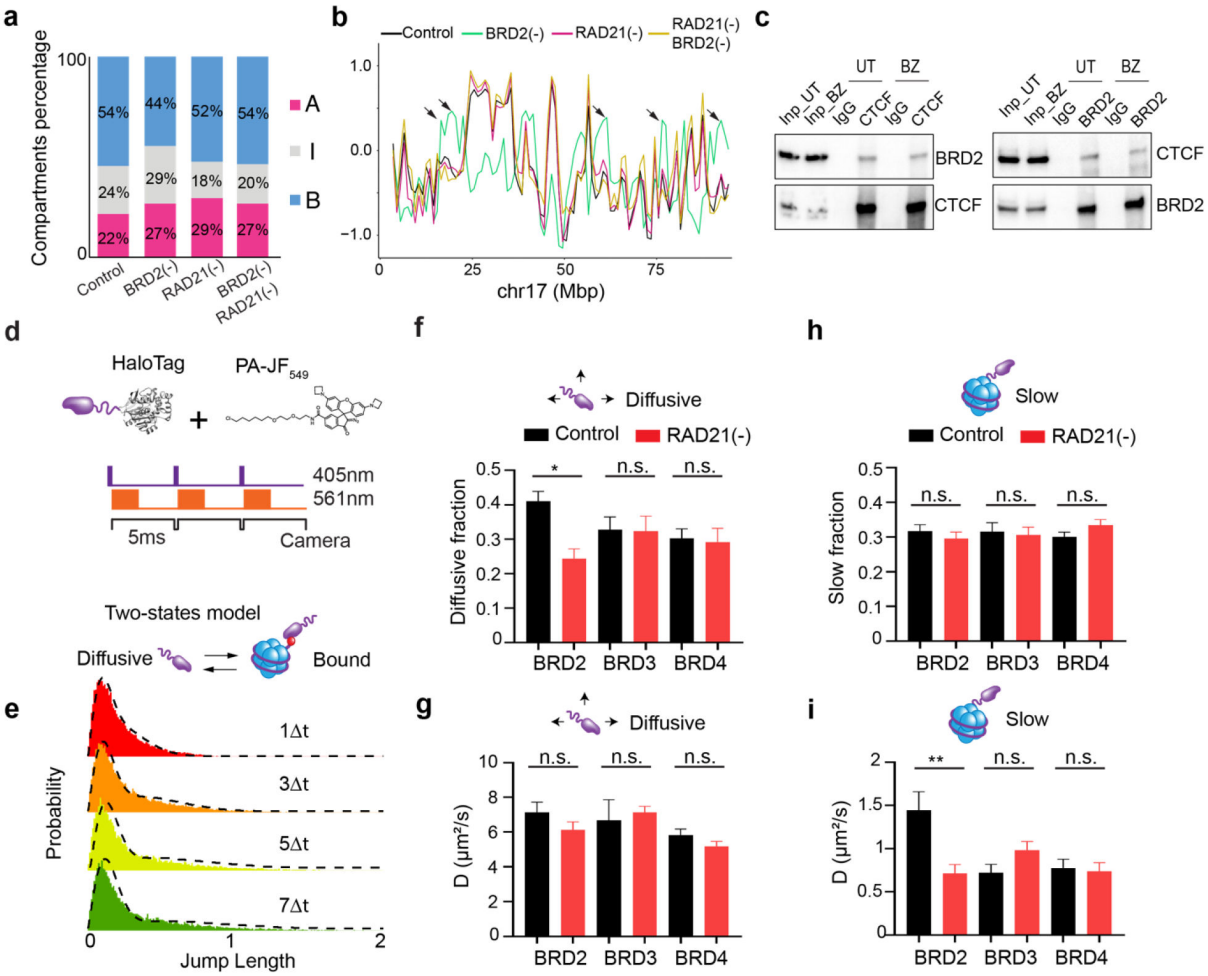
BRD2 interplays with Cohesin. Related to Fig.3. **(a)** Quantification of compartmental changes after BRD2 and RAD21 depletion individually or in combination. A, active compartments; I, intermediated mixed compartments; B, inactive compartments. The mouse genome is binned into 25480 segments (1Mbp bin size) and the percentage of each fraction is shown. See details of compartment analysis in the method section. **(b**) Browser track view of the eigenvector values for compartmental score in various perturbation conditions on chromosome 17. The genomic regions containing B to A switches after BRD2 depletion (green line) are highlighted with black arrows. **(c**) Endogenous Co-immunoprecipitation between BRD2 and CTCF in mouse ESCs. Benzonase (BZ) treatment did not abrogate the BRD2-CTCF interaction as compared to untreated control (UT). **(d**) (Upper panel) Schematic of genome engineering of BET proteins with HaloTag and labeled with Janelia photoactivatable fluorophore PA-JF_549_ for single molecule tracking. (Lower panel) Illustration of stroboscopic single molecule imaging of BET protein dynamics in live cells. After activation by a 405 nm pulse, single BET molecules were excited by 561 nm laser pulse (2 ms) to suppress motion blurring and images were captured with ~5ms exposure times. **(e**) The probability distribution function of jump length was fit over multiple camera integration time scales by the two-state model estimated by Spot-On program (See methods). The two-state diffusive vs bound model does not well account for the dynamics of BRD2 or BRD3/4 (not shown) as compared to the three-state model in Fig.3c. **(f-g**) The diffusive fraction (**f**) and its diffusion coefficient (**g**) analysis for BRD2, BRD3 and BRD4 from SMT analysis after Cohesin depletion. Cohesin depletion significantly decreased the diffusive fraction of BRD2 accompanied with an increase of the chromatin bound fraction. The number of cells analyzed are the same as Fig.3d-e. **(h-i**) The slow bound fraction (**h**) and its diffusion coefficient (**i**) analysis for BRD2, BRD3 and BRD4 from SMT analysis after Cohesin depletion. Cohesin depletion also significantly decreased the diffusion coefficient of slow bound BRD2. The number of cells analyzed are the same as Fig.3d-e.

**Extended Data Fig.9 ∣:**
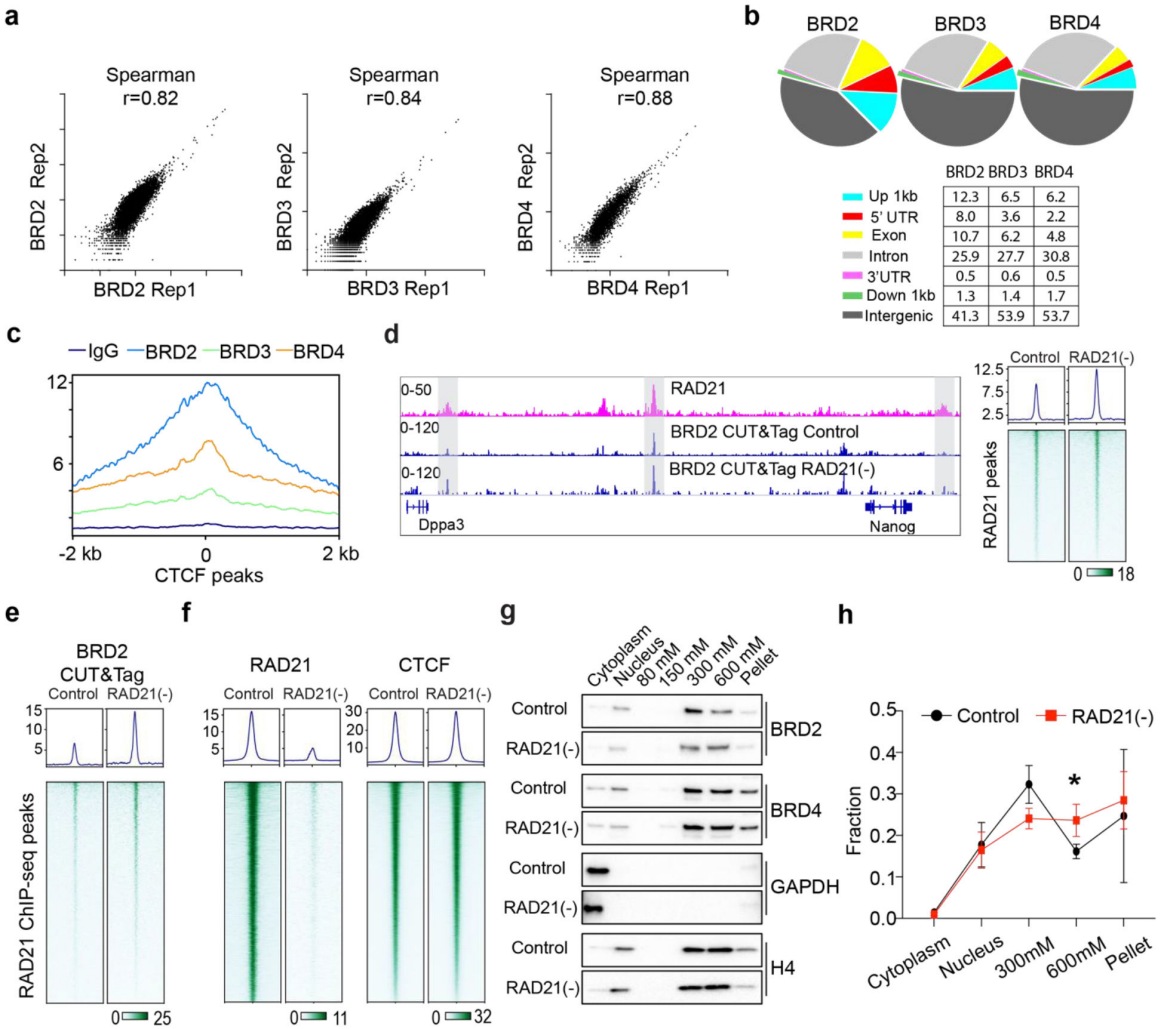
Genomic and biochemical analysis of BET family proteins. Related to Fig.3. **(a**) Scatter plot of two biological replicates of ChIP-seq signal for BRD2, BRD3 and BRD4. Ranked spearman correlation coefficient is shown above each plot. **(b**) Pie diagram of different genomic binding features of BRD2, BRD3 and BRD4 extracted from their respective ChIP-seq peak signal. The percentage of BET proteins binding to different genome features are summarized in the table on the bottom. **(c)** Enrichment of BET protein ChIP-seq signal at CTCF sites was performed using k-means clustering (n=2, enriched and non-enriched). BRD2 has the highest enrichment profile among BET proteins at CTCF sites at the binding-enriched cluster. **(d)** (Left panel) Representative genome browser track of Cohesin ChIP-seq (magenta) and BRD2 CUT&Tag (blue) before and after Cohesin depletion (RAD21(−)). Shaded area demonstrates the increased BRD2 signal at Cohesin binding peaks. (Right panel) BRD2 enrichment and heatmap analysis by CUT&Tag (Cell Signaling, 5848S) at Cohesin ChIP-seq peaks before and after Cohesin depletion. **(e)** Genome wide heatmap analysis of BRD2 CUT&Tag enrichment at Cohesin ChIP-seq peaks before and after Cohesin depletion by using a different antibody (Bethyl laboratories, A302-583A). **(f)** ChIP-seq analysis of RAD21 and CTCF after 6 hours Cohesin depletion. Shown are the enrichment profile (upper panel) and heatmap (lower panel) of each protein over its binding peaks. **(g**) Chromatin fractionation assay of BRD2 and BRD4 in non-treated Control and RAD21 depletion conditions after different salt extraction of ESC nuclei followed by western blot analysis. GAPDH and Histone H4 was used as marker for the cytoplasmic and chromatin bound fraction, respectively. In control cells, BRD2 is preferentially extracted from the 300mM NaCl concentration. After Cohesin depletion, more BRD2 is extracted from the 600mM NaCl concentration. **(h**) Summary of the relative BRD2 enrichment in different chromatin fractionations before and after RAD21 depletion from 4 replicates. Each fraction is normalized to histone H4 abundance. * indicates p < 0.05 by non-parametric Mann-Whitney test. Error bar represents standard deviation. The non-parametric Mann-Whitney U test was used for statistical testing. *, p < 0.05.

**Fig.3 ∣:**
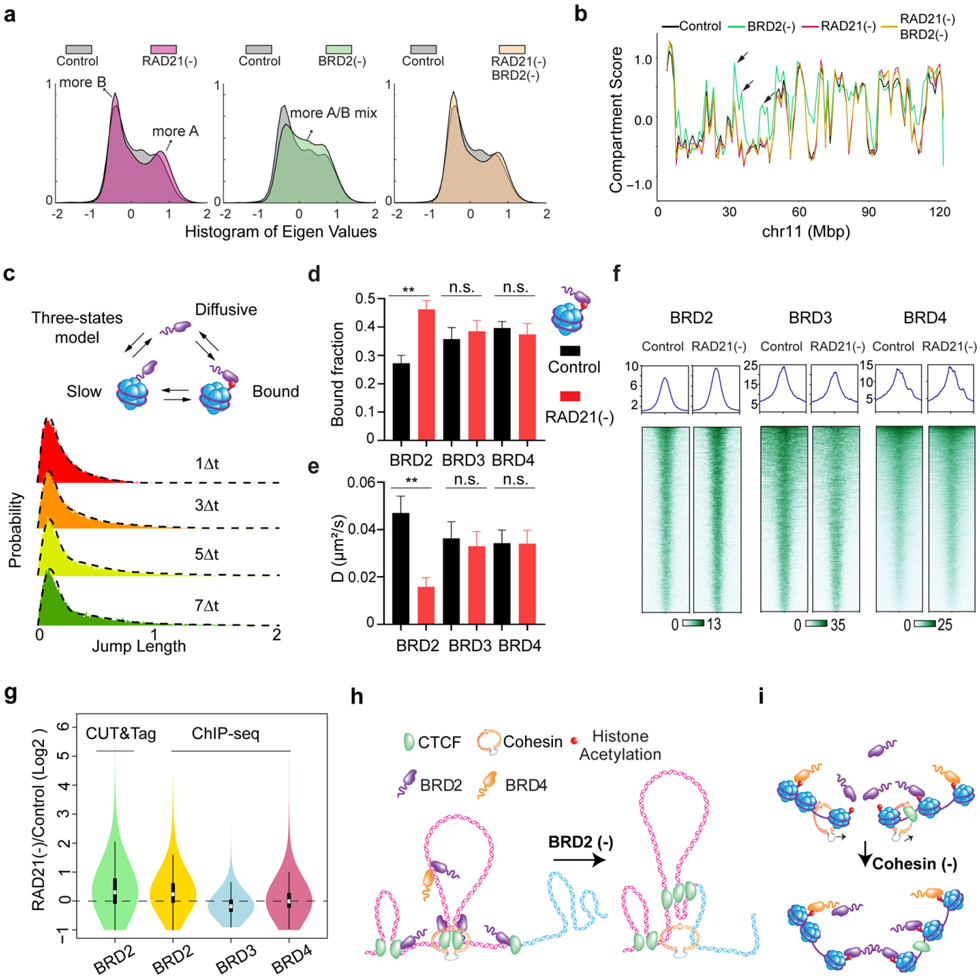
BRD2 interplays with Cohesin to safeguard active compartments. **(a)** Histogram of eigenvector values from the Pearson’s correlation matrix of single BRD2 or RAD21 depletion and dual RAD21/BRD2 depletion for 6 hours compared to untreated Control from Micro-C experiments. BRD2 depletion increases the switching of B to A compartments. **(b**) Browser track view of the eigenvector values for compartmental scores in various perturbation conditions in chromosomes 11 (Chr11). The genomic regions containing B to A switches after BRD2 depletion (green line) are highlighted with black arrows. **(c)** Live cell single molecule tracking (SMT) of BET proteins by stroboscopic imaging. The jump length fitting of BRD2 dynamics is best described by a three-state model: diffusive, slow (likely transient, non-specific collision) and bound (likely stably bound to cognate sites). The probability distribution function of jump length or single molecule displacement was fit over multiple camera integration time scales. The same three-state model applies to BRD3 or BRD4 (data not shown). **(d-e**) The chromatin bound fraction **(d)** and diffusion coefficient D **(e)** of BRD2, BRD3 and BRD4 before and after 6 hours of RAD21 depletion were quantified from SMT experiments. The number of cells analyzed are n=17 and n=19 for control and RAD21 depletion for BRD2 SMT, n=17 and n=17 for control and RAD21 depletion for BRD3 SMT, and n=17 and n=19 for control and RAD21 depletion for BRD4 SMT. **(f)** ChIP-seq analysis of BRD2, BRD3 and BRD4 after 6 hours Cohesin depletion. Shown are the enrichment profile (upper panel) and heatmap (lower panel) of each protein over its binding peaks. **(g**) A violin plot showing the log2 fold change of BRD2 CUT&Tag and BRD2/3/4 ChIP-seq intensity at corresponding peaks. Both BRD2 CUT&Tag and BRD2 ChIP-seq show preferential increase at BRD2 ChIP-seq peak regions compared to BRD3/BRD4. **(h)** Putative model of B to A compartmental switch after removing BRD2. The active A compartment is colored in pink whereas the inactive B compartment in cyan. BRD2 molecules bind to the active A compartment enriched with CTCF and acetylated nucleosomes. BRD2 depletion weakens the boundary resulting in more Cohesin translocation into the neighboring inactive segments and more A/B mixing. **(i)** Putative model showing the enhanced chromatin binding of BRD2 associated with an increased spatial clustering of ACDs upon Cohesin loss. For simplicity, two small segments containing acetylated nucleosomes are shown. The non-parametric Mann-Whitney U test was used for statistical testing. **, p < 0.01; n.s., not significant.

The ability of BRD2 to mix accessible chromatin in the absence of Cohesin prompted us to investigate the role of BRD2 in the presence of Cohesin. We performed Micro-C experiments and found that BRD2 depletion alone caused considerable switching of inactive compartments to intermediate or active ones and, interestingly, these compartmental switches are dependent on Cohesin (Fig.3a-b, Extended Data Fig.8a-b). These results are consistent with previous reports that BRD2 selectively associates with CTCF to protect architectural boundaries ^53,54^. We also found that endogenous BRD2 complexes with CTCF independent of nucleic acids in mouse ESCs (Fig.8c) and that BRD2 has the highest enrichment at CTCF binding sites among BET family proteins (Extended Data Fig.9a-c).

Next, we investigated how Cohesin loss would affect the binding of BET proteins to chromatin by single molecule tracking (SMT) (Extended Data Fig.8d). Fast stroboscopic SMT revealed that the diffusion and binding dynamics of BET proteins is best described by three molecular states toggling between rapid diffusion (~40%), tight chromatin engagement (~30%) and transient chromatin binding (~30%) (Fig.3c, Extended Data Fig.8e). Interestingly, we found that Cohesin loss increased the tight chromatin engagement fraction while decreased the diffusive fraction of BRD2 but not of BRD3 or BRD4 (Fig.3d, Extended Data Fig.8f and 8h). Correspondingly, BRD2 diffusion coefficients of both tight and transient chromatin-engaged fractions were reduced, suggesting a tighter association of BRD2 to chromatin upon Cohesin loss (Fig.3e, Extended Data Fig.8g and 8i). Consistent with these findings, ChIP-seq and CUT&Tag revealed that genome-wide binding of BRD2 but not BRD4 or BRD3 was modestly enhanced after Cohesin loss (Fig.3f-g, Extended Data Fig.9d-f). In support of these imaging and genomic results, biochemical fractionation experiments showed that BRD2 was preferentially eluted at higher salt concentrations from Cohesin-depleted chromatin than from untreated control (Extended Data Fig.9g-h). Taken together, these observations suggested that BRD2 is the effector among the BET family proteins that reacts to Cohesin loss by binding more to active chromatin. These results begin suggesting a model where Cohesin antagonizes BRD2 binding to chromatin and counteracts the ability of BRD2 to promote interactions between ACDs. Thus, an intricate interplay between BRD2 and Cohesin might exist to maintain the finely balanced genome compartmentalization in the nucleus (Fig.3h-i).

### BRD4 competes with BRD2 and blocks its activities in genome organization

**Extended Data Fig.10 ∣:**
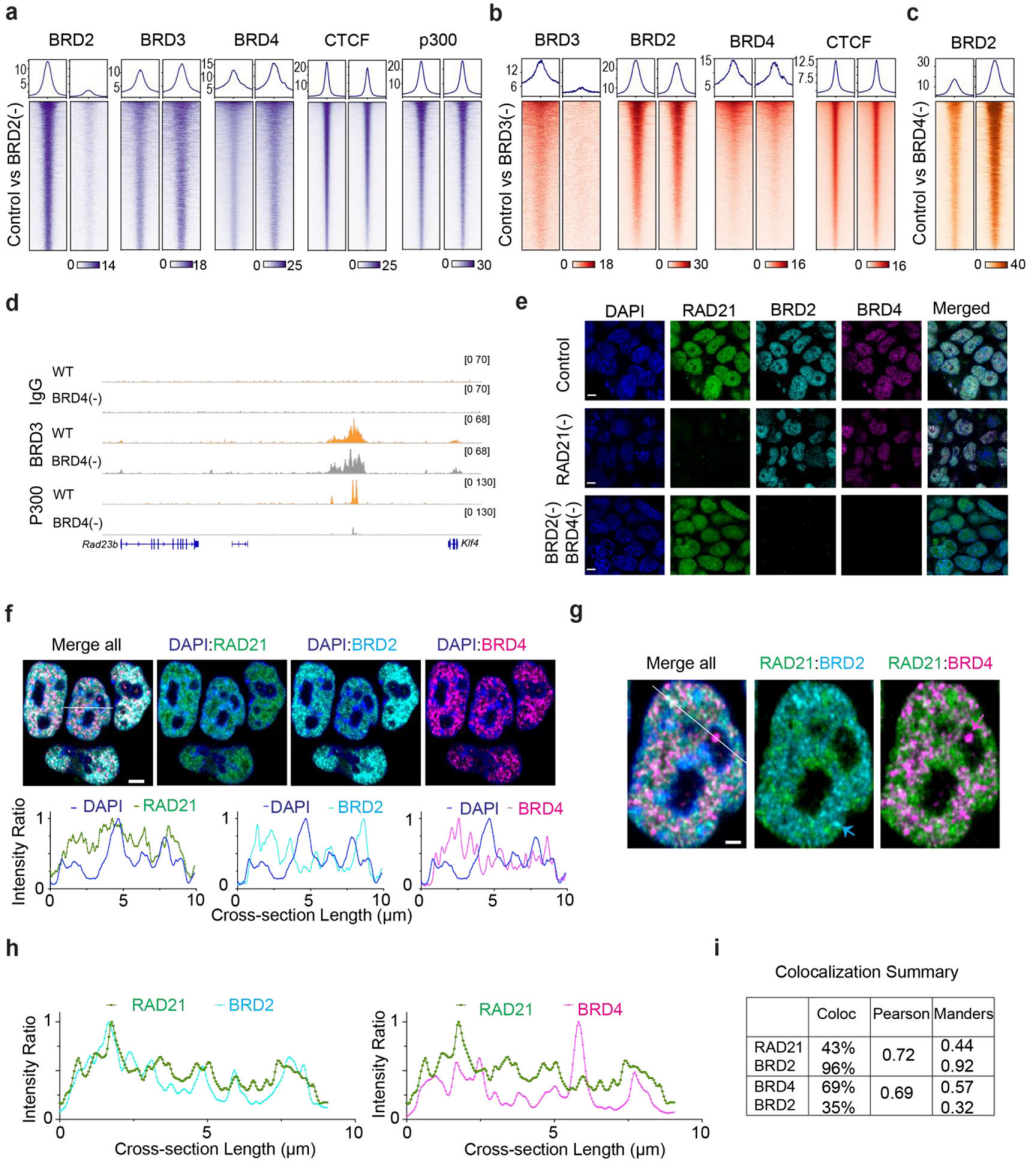
BRD4 regulates BRD2 chromatin binding and function. Related to Fig.4. **(a)** Enrichment profile and heatmap of BRD2/3/4, CTCF, P300 ChIP-seq signal at their respective binding peaks before and after BRD2 depletion. **(b)** Enrichment profile and heatmap of ChIP-seq signal of BRD2, BRD3, BRD4 and CTCF at their cognate binding peaks after 6 hours of BRD3 depletion. **(c)** Heatmap of BRD2 binding signal specifically at BRD2/BRD4 co-bound regions before and after acute BRD4 depletion for 6 hours. **(d**) Representative genomic tracks (from integrated genomics viewer) of IgG, BRD3 and p300 after BRD4 depletion. BRD4 depletion markedly reduced P300 binding. **(e)** Validating the specificity of fluorescence signal or antibodies used for immunofluorescence after acute depletion of RAD21 or BRD2/BRD4. The RAD21-mAID-eGFP : BRD2-HA-dTAG : BRD4-Halo-dTAG triple degron mESC line was used in this study. RAD21 was detected by covalently tagged eGFP signal. BRD2 was detected by mouse monoclonal anti-HA antibody and goat-anti-mouse Alexa568 secondary antibody. BRD4 was detected by rabbit BRD4 primary antibody and goat-anti-rabbit Alexa647 secondary antibody. To validate the fluorescence signal specificity, RAD21 and BRD2/BRD4 were depleted by adding auxin (500μM) or dTAG13 (100nM) for 6 hours, respectively. **(f)** (Upper panel) Representative images of spatial distribution of RAD21, BRD2 and BRD4 signal. (Lower panel) Fluorescent Intensity profile of RAD21(green), BRD2 (cyan) and BRD4 (magenta) relative to DAPI (blue). RAD21 is enriched at both DAPI high and low regions whereas BRD2 and BRD4 are enriched at DAPI low regions. Scale bar, 3μm. **(g)** Representative single cell view of Cohesin (RAD21, green), BRD2 (cyan) and BRD4 (magenta) in their merged view. The while line indicates the cross-sectional line to analyze the fluorescent intensity profile. The cyan arrow indicates the BRD2 puncta showing little colocalization with BRD4. The magenta arrow indicates the BRD4 puncta poorly colocalized with BRD2. Scale bar, 1μm. **(h)** Fluorescence intensity profile of Cohesin (RAD21, green), BRD2(cyan) and BRD4 (magenta) along the while line in **(g).** **(i)** Whole nucleus 3D voxel-to-voxel correlation summary of RAD21 vs BRD2 and BRD4 vs BRD2 as shown in Fig.4c. The percentage of voxel colocalization of protein pair, the Pearson’s coefficient, and the Manders’ coefficient are computed and summarized.

**Extended Data Fig.11 ∣:**
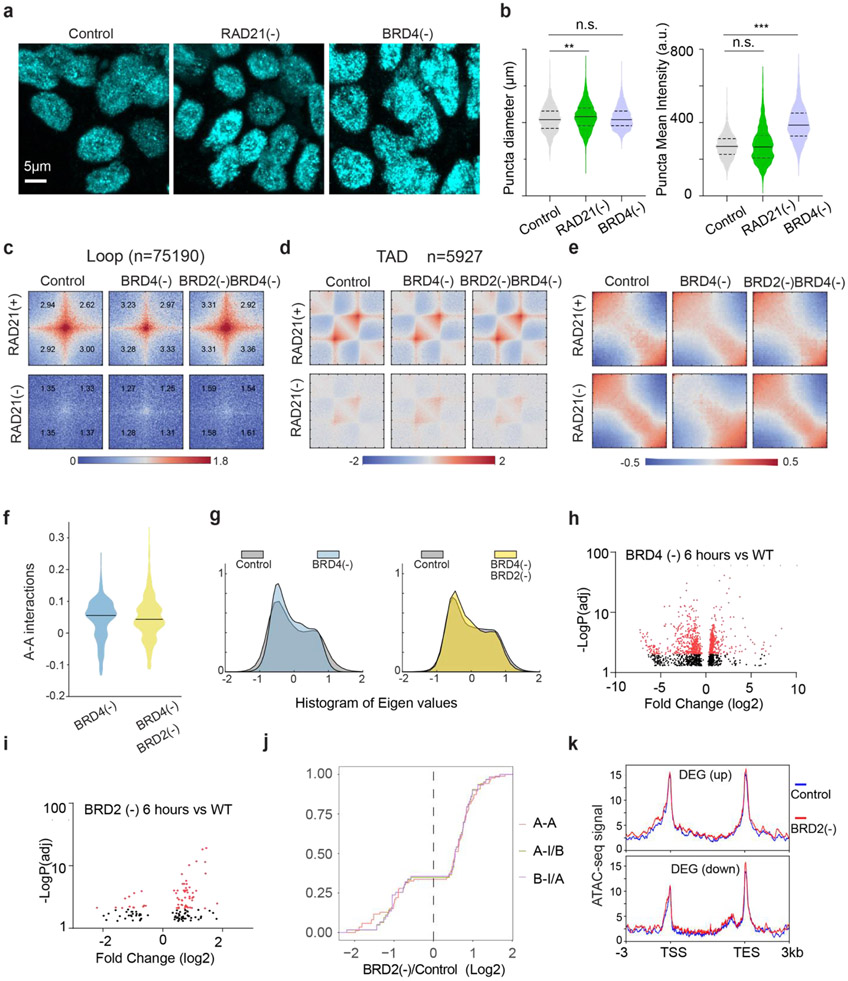
BRD4 regulates BRD2 chromatin binding and function. Related to Fig.4. **(a)** Representative images of BRD2 immunofluorescence before and after acute RAD21 (500μM auxin 6 hours) or BRD4 depletion (100nM dTAG13, 6 hours) in the dual RAD21-mAID-eGFP and BRD4-Halo-dTAG mESC line. BRD2 was detected by anti-BRD2 primary antibody and goat anti-rabbit Alexa568 secondary antibody. **(b)** Quantification of BRD2 puncta size (left panel) and puncta mean intensity (right panel) in control (n = 1502), RAD21 depletion (n = 2314), BRD4 depletion (n = 2861) conditions, n represent the number of BRD2 puncta analyzed. **(c-e**) Micro-C analysis of loops (**c**), TADs (**d)** and compartments (**e**) after BRD4 depletion or dual BRD2/BRD4 depletion in the presence or absence of Cohesin. In **e**, saddle plots of compartmental interactions are shown. **(f-g**) Micro-C analysis of active compartmental interactions (A-A interactions) (**f**) and histogram of eigenvalues from the Pearson’s correlation matrix (**g**) for BRD4 depletion alone or dual BRD4/BRD2 depletion. **(h-i**) RNA-seq analysis of differentially expressed genes (DEGs) after BRD4 **(h)** or BRD2 **(i)** depletion. Volcano plot of significantly (p<0.05) expressed genes after 6 hours depletion of BRD4 or BRD2 are shown. Red dots indicate significant expressed genes with p<0.01. False positive rate adjusted p value in the -log10 form are shown in the y axis. We detected 133 DEGs for BRD2 depletion and 1579 genes for BRD4 depletion. **(j)** Cumulative distribution function of differentially expressed genes (DEGs) after BRD2 depletion over control relative to compartmental switches. A-A indicates no switch within the A compartment. A-I/B, switches from A to intermediate (I) or B compartment. B-I/A, switches from B to I or A compartment. The overlapping curves suggest no obvious association of DEGs with compartmental changes. **(k)** Profiling of chromatin accessibility by ATAC-seq before and after acute BRD2 depletion (6 hours) over DEGs (upregulated and downregulated in Fig**.(i)**).

**Fig.4 ∣:**
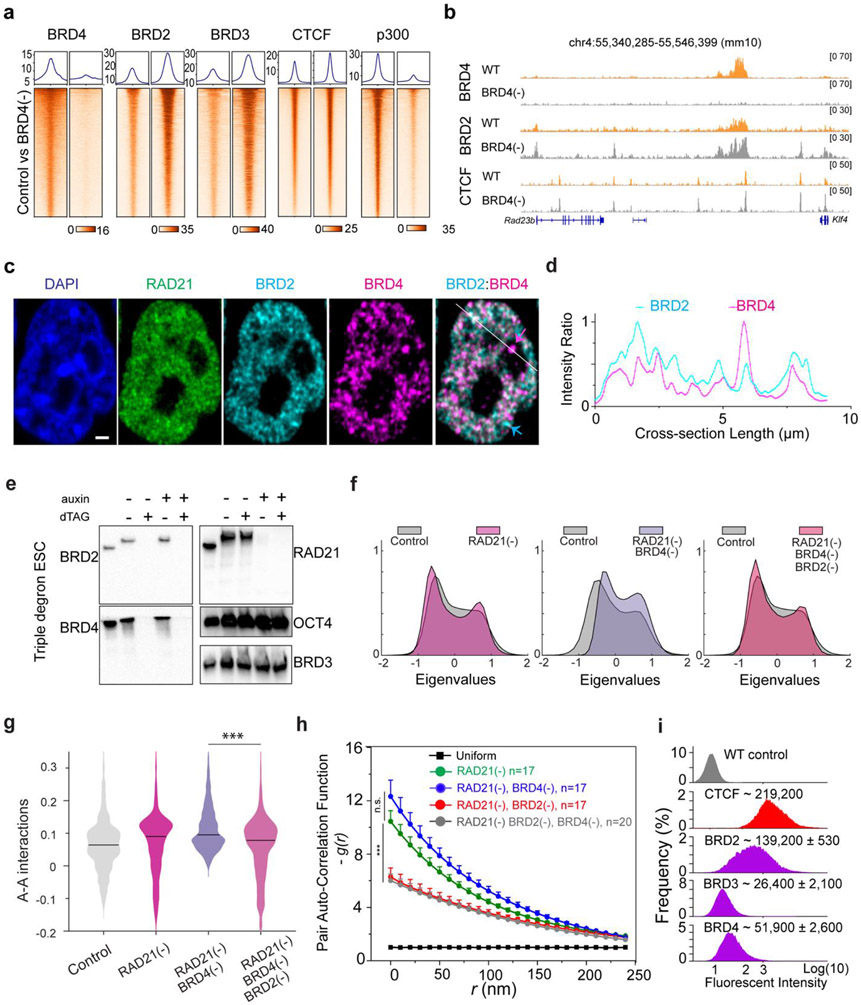
BRD4 antagonizes the role of BRD2 in genome organization. **(a)** Enrichment profile and heatmap of BRD2/3/4, CTCF, P300 ChIP-seq signal at their respective binding peaks before and after BRD4 depletion. **(b)** Representative genomic tracks (from integrated genomics viewer) of ChIP-seq signal of BRD2, BRD4 or CTCF after acute depletion of BRD4 for 6 hours. **(c)** Representative single cell view of Cohesin (RAD21, green), BRD2 (cyan) and BRD4 (magenta) and merged BRD2/BRD4. The cyan arrow indicates the BRD2 puncta showing little colocalization with BRD4. The magenta arrow indicates the BRD4 puncta poorly colocalized with BRD2. The white line indicates the region to profile the fluorescent intensity. Scale bar, 1μm. **(d)** Fluorescence intensity profile of BRD2(cyan) and BRD4 (magenta) along the while line in **(c).** The relative intensity ratio is plotted. **(e)** WB analysis of the *Rad21-AID : Brd4-dTAG : Brd2-dTAG* triple edited ESC line. dTAG13 treatment (100nM, 6 hours) simultaneously depletes both BRD2 and BRD4 whereas auxin treatment (100μM, 6 hours) orthogonally depletes RAD21. **(f)** Histogram of eigenvector values from the Pearson’s correlation matrix of single RAD21 depletion, dual RAD21/BRD4 or triple RAD21/BRD4/BRD2 depletion for 6 hours compared to untreated Control from Micro-C experiments. **(g)** Quantification of the digitalized A-A compartmental interactions (log10 value of observed/expected) after single RAD21 depletion, dual RAD21/BRD4 depletion or triple RAD21/BRD2/BRD4 depletion for 6 hours from Micro-C experiments. Triple RAD21/BRD4/BRD2 depletion reduced the enhanced compartmentalization after the dual Cohesin/BRD4 depletion. The black solid line in each violin plot represents the median value. **(h)** Degradation of BRD2 significantly reduced the accessible chromatin clustering after dual BRD4/RAD21 depletion. *g(r)* curves were plotted for indicated conditions. The non-parametric two-sided Mann-Whitney U test was used for statistical testing. **(i)** Biallelic knock-in of HaloTag into endogenous BET family genes enable accurate quantification of individual BET family protein copy number by CTCF-calibrated flow cytometry in live cells. The mean and standard deviation of the quantified copy number from two biological experiments are shown above each plot. The non-parametric Mann-Whitney U test was used for statistical testing. ***, p < 0.001; n.s., not significant.

The unexpected role of BRD2 in compartmentalizing the accessible genome prompted us to examine the interrelationship among BET family proteins. We first performed ChIP-seq experiments after acute depleting individual BET family member. Although acute loss of one BET protein did not significantly alter the mRNA or protein levels of other BET proteins (Fig.2b, Extended Data Fig.6j, Fig.6m, Supplementary Tables 1-2), we noticed that BRD2 depletion resulted in enhanced binding of BRD4 and BRD3 to chromatin (Extended Data Fig.10a). Conversely, BRD4 loss considerably increased the chromatin binding of BRD2 and BRD3 (Fig.4a-b; Extended Data Fig.10c-d). High resolution imaging also shows that BRD2 and BRD4 have co-localized yet non-overlapping nuclear distributions (Fig.4c-d, Extended Data Fig.10e-i). BRD4 depletion appears to increase the fluorescent intensity of BRD2 puncta (Extended Data Fig.11a-b). These genomic and imaging results suggested that BET proteins might compete rather than synergize with each other to bind to a common set of targets likely via their evolutionarily conserved double bromodomain. Interestingly, the increased BRD2 binding after BRD4 loss is also associated with a stronger CTCF binding (Fig.4a-b), consistent with the reported role of BRD2 in potentiating CTCF-marked architectural boundaries^53^.

We realized that the elevated BRD2 chromatin binding induced by BRD4 depletion could serve as a useful system to further test the role of BRD2 in genome organization. Specifically, we expected that BRD4 depletion would increase interactions between active compartments upon Cohesin loss as more BRD2 molecules were permitted to bind to chromatin. Consistent with this prediction, Micro-C experiments showed that BRD4 depletion considerably increased interaction frequencies between active compartments after Cohesin removal (Fig.4e-g; Extended Data Fig.11c-g). To test whether increased inter-compartmental chromatin contacts are mediated by BRD2, we generated a triple Cohesin/BRD4/BRD2 inducible degradation cell line, which allowed us to robustly deplete both BRD4 and BRD2 (Fig.4e). Micro-C experiments showed that BRD2 depletion reversed the increased inter-compartmental chromatin contacts (Fig.4f-g). 3D ATAC-PALM imaging also demonstrated that additional BRD2 depletion significantly reduced the spatial clustering of accessible chromatin after depletion of Cohesin and BRD4 (Fig.4h). These results suggested that enhanced compartmental contacts induced by BRD4 depletion in the absence of Cohesin are dependent on BRD2.

Taken together, these genomic and imaging experiments supported a functional role of BRD2 in regulating the accessible genome topology and implied a division of labor for BRD2 and BRD4 to govern distinct regulatory processes in the accessible genome. Consistent with this notion, we found that BRD4 but not BRD2 depletion significantly reduced the binding of transcriptional coactivator P300 to chromatin (Fig.4a, Extended Data Fig.10a, 10d). Moreover, RNA-seq experiments revealed that acute BRD4 depletion affected the expression of a much larger pool (~thousands) of genes than BRD2 depletion (~100 genes) (Extended Data Fig.11h-i, Supplementary Table 1-2). Interestingly, the small set of differentially expressed genes after BRD2 depletion does not appear to correlate with compartmental switches or changes in chromatin accessibility (Extended Data Fig.11j-k). Finally, we found that BRD3 depletion did not appear to significantly impact BRD2, BRD4 or CTCF binding to chromatin (Extended Data Fig.10b), possibly due to the fact that BRD3 is expressed at significantly lower levels than BRD2 and BRD4 in ESCs (Fig.4i).

### Polymer modeling incorporating loop extrusion and chromatin state-specific affinity interactions

**Extended Data Fig.12 ∣:**
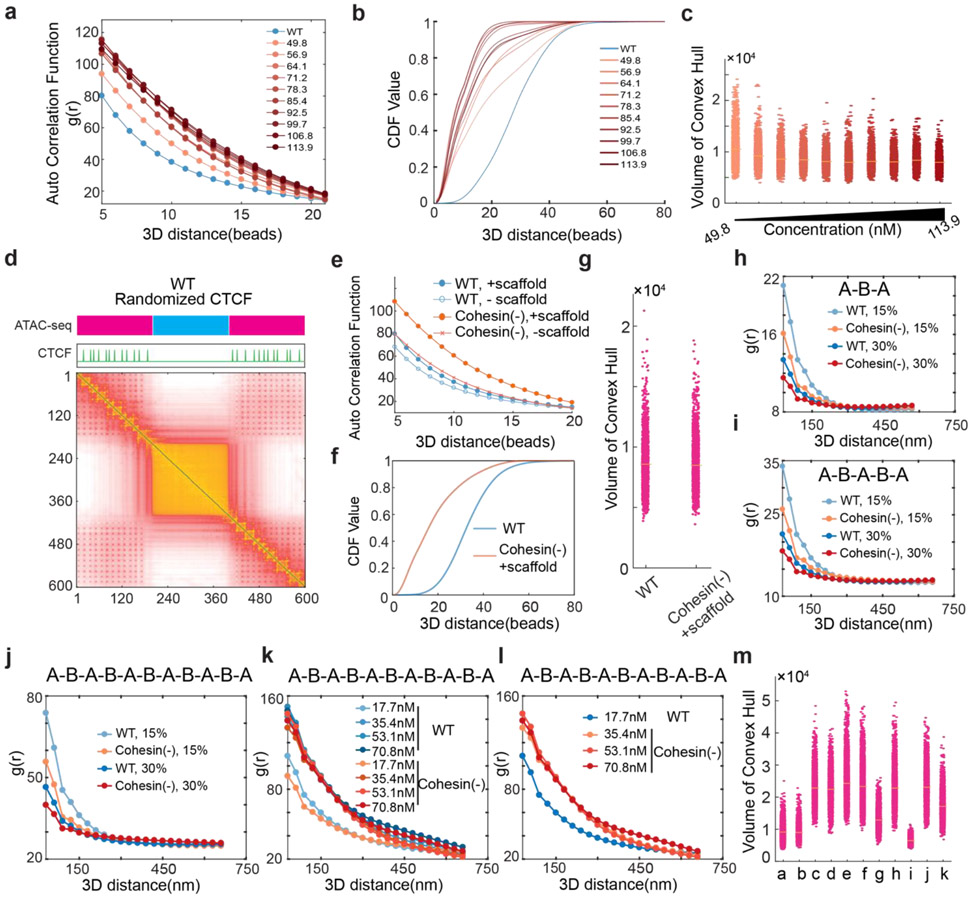
Robustness of the polymer model that incorporates both loop extrusion and scaffold protein-mediated chromatin interactions. Related to Fig.5. **(a-c)** Testing the robustness of the polymer model by varying the scaffold protein concentrations from 21.3nM (WT) to 113.9nM. Once passing a certain threshold (56.9nM), simulations show minimal impact of scaffold concentrations on the pair auto-correlation function *g(r)* (**a**), the cumulative distribution function (CDF) of 3D distance between neighboring active segments (**b**), and distributions for the volume of the convex hull for individual ATAC-rich segment (**c**). The yellow bar represents mean value. **(d-g)** Testing the robustness of the polymer model by changing the distribution of CTCF sites on the ATAC-rich segment. (**d**) Contact probability map for the WT condition in which the CTCF beads are randomized. (**e**) Simulations here also demonstrate that randomized CTCF site distribution on active chromatin does not change the pair auto-correlation function *g(r)* as shown in Fig.7b. (**f**) CDF of 3D distance between neighboring active segments after Cohesin loss with randomized CTCF sites. (**g**) distributions for the volume of the convex hull for individual ATAC-rich segments after CTCF sites randomization. The yellow bar represents mean value. **(h-j**) Simulation results are robust with respect to the varying model setups. Pair auto correlation function *g(r)* for system with different sizes A-B-A (**h**), A-B-A-B-A (**i**), A-B-A-B-A-B-A-B-A-B-A (**j**) and chromatin volume fraction (15% with lighter color, 30% with denser color) for wild type (WT, in blue) and Cohesin depletion (Cohesin (−), in red). **(k)** *g(r)* showing various protein concentration for WT (blue) and Cohesin depletion (red) of system A-B-A-B-A-B-A-B-A-B-A with 15% chromatin volume fraction. Protein concentration from low to high are marked with colors from light to dense. **(l)** Testing the robustness of the polymer model by varying system setup from A-B-A to A-B-A-B-A-B-A-B-A-B-A with chromatin volume fraction of 15%, and various protein concentration. *g(r)* showing various protein concentration for WT (blue) and Cohesin depletion (red) are marked with colors from light to dense to represent protein concentration from low to high. **(m)** The protein-DNA interaction, multivalency, and optimal protein-protein interactions are required to maintain the ATAC-rich segment configuration upon Cohesin removal. Simulation for the volume of the convex hull of ATAC-rich segments under different perturbation conditions. The panels from left to right show the results of (a) Wild type, and Cohesin depletion with (b) current model setup, (c) no protein-DNA interaction, (d) no protein-protein interaction, (e) scaffold protein with only one interaction segment (lacking multivalency), (f) reduced protein-protein interaction strength (2kT), (g) increased protein-protein interaction strength (4kT), (h) reduced protein-DNA interaction strength (2kT), (i) increased protein-DNA interaction strength (6kT), (j) reduced protein-protein interaction strength (2kT) with higher protein concentration, (k) increased protein-protein interaction strength (4kT) with low protein concentration. k is the Boltzmann constant and T is the temperature. See more details in the Robustness of simulation parameters in the methods section.

**Fig.5 ∣:**
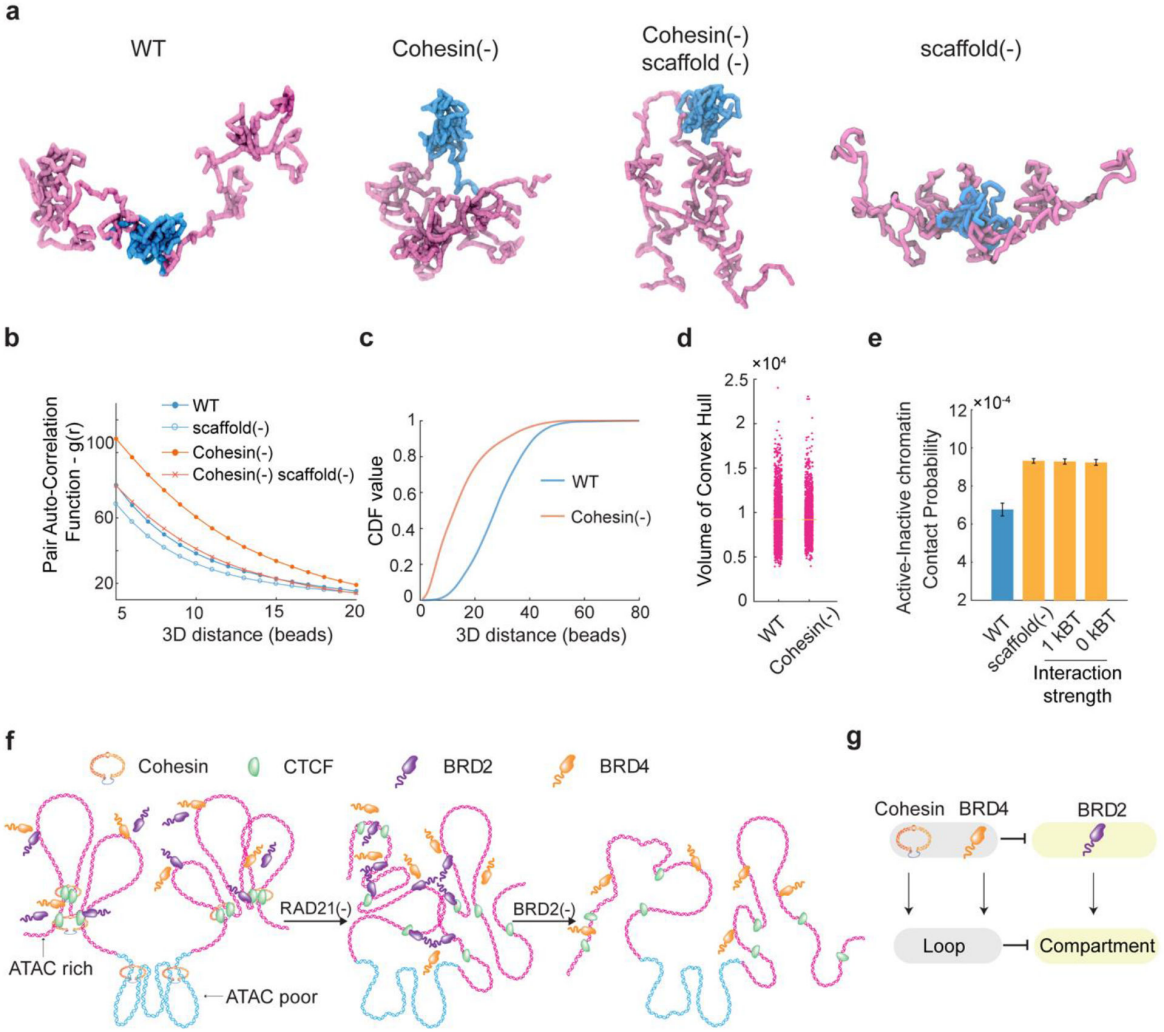
A revised polymer model recapitulates experimental observations. **(a)** Representative chromatin conformation snapshots from polymer simulation incorporating both loop extrusion and scaffold protein-mediated interactions. Simulation snapshots represent wild type (WT, incorporating Cohesin, CTCF and scaffold protein), Cohesin depletion (−), scaffold depletion (−) and dual Cohesin and scaffold depletion. See details in the Polymer Modeling in the method section. **(b)** Polymer simulation integrating both loop extrusion and scaffold protein-mediated dynamic protein-chromatin and protein-protein interactions reproduces enhanced clustering of ACDs upon Cohesin depletion. The pair auto-correlation function *g*(*r*) for active chromatin beads from all recorded polymer conformations were plotted for WT and Cohesin depletion in which the scaffold protein is present or depleted (−). Removing the scaffold protein decreases the *g*(*r*) as observed in Fig.2c and Extended Data Fig.4d. **(c)** The cumulative distribution function (CDF) value as a function of the 3D loci pair distance from the center of neighboring ATAC-rich segments were extracted in WT (blue curve) and Cohesin depletion (−) plus scaffold protein conditions in which both loop extrusion and scaffold protein dynamic interaction are incorporated in the revised polymer model. **(d)** Polymer model predictions of the segment volume of ATAC-rich segments under WT and Cohesin depletion (−) plus scaffold protein conditions. The new polymer model recapitulates experimental observations of the volume changes in Extended Data Fig.3a-b. **(e)** Average contact probability between ATAC-rich regions and ATAC-poor regions for WT, scaffold protein depletion, WT with protein-protein interaction reduced from 2.5k_B_T to 1.0k_B_T (non-specific) and WT with protein-protein interaction depleted (0k_B_T) calculated from simulations. Error bar indicates the range of average contact probability from two ATAC-rich regions from both sides with the ATAC-poor regions in the middle. k_B_, Boltzmann constant; T, temperature. **(f**) Schematic of putative chromatin configurational changes under WT (left), Cohesin depletion (middle) or dual Cohesin/BRD2 depletion (right) conditions. ATAC-rich and ATAC-poor segments are shown in pink and cyan color, respectively. Although Cohesin loss eliminates Cohesin-dependent loops, BRD2 (and others) orchestrates extensive protein-protein and protein-chromatin interactions that lead to the enhanced chromatin interactions between ATAC-rich segments and largely unchanged 3D volume of individual ATAC-rich segments. Further BRD2 depletion mitigates interactions between active chromatin segments and decompacts individual ATAC-rich segments. **(g**) Summary model. Cohesin is the master regulator of chromatin loops. BRD4 could associate with NIPBL to enhance chromatin loop formation ^70^. Both BRD4 and Cohesin could suppress BRD2 binding to chromatin, which in turn antagonize chromatin compartmentalization in euchromatin.

Our imaging and genomic results associated with BRD2 prompted us to revise the polymer simulation by incorporating a scaffold protein that preferentially binds to active chromatin and mediates multivalent affinity interactions (Fig.5a, also see more details in the Polymer Modeling section in Methods). We found that, by increasing the scaffold protein concentrations binding to chromatin upon Cohesin depletion just as we observed for BRD2 (Fig.3d-g, Extended Data Fig.9g-h), the revised polymer simulation was able to qualitatively reproduce the general trends observed in our imaging experiments, including: i) enhanced clustering of accessible chromatin and shortening of distances between active domains *in cis* upon Cohesin removal, ii) reduced clustering of accessible chromatin after JQ1/1,6-HD treatment or BRD2 depletion, and iii) lack of volume changes in active domains after Cohesin depletion (Fig.5b-d). Moreover, in the presence of Cohesin, removing the scaffold protein or its multivalent protein-protein interactions was found to increase the contact probability between neighboring active and inactive segments (Fig.5e), in agreement with the compartmental switches observed by Micro-C (Fig.3a-b, Extended Data Fig.8a-b). The trends generated from the polymer model are reproducible under a wide range of scaffold protein concentrations and domain configurations (Extended Data Fig.12a-l), confirming the generality and robustness of the model. This minimalist model delineated the possibility that Cohesin-mediated loop extrusion competes with chromatin-state specific affinity interactions mediated by multivalent binders such as BRD2, which play a key role in compartmentalizing the accessible genome (Fig.6f-g). In agreement with our Micro-C results that both double bromodomain and LCD are required for BRD2 mediated compartmentalization (Fig.2h-i, Extended Data Fig.7f-k), protein-DNA and protein-protein interactions and multivalency are all required to recapitulate the experimental results (Extended Data Fig.12m).

## Discussion

In the past decade, the Cohesin loop extrusion model has been successful in explaining several key aspects of nuclear topology. However, results from multiple studies in conjunction with our observations here suggest that Cohesin-independent mechanism(s) may also control the spatial patterning of chromatin. Although discussed at the theoretical level ^55-57^, the precise nature underlying these mechanisms however remains largely unclear.

Here, by using a combination of chemical and genetic perturbations, super resolution microscopy and genomics, we identified the BET family protein BRD2 as a regulator that facilitates chromatin contacts, mixes and compacts active chromatin in the absence of Cohesin. Interestingly, BRD2 is the most abundant BET protein and the only protein in the family that reacts to Cohesin loss by interacting more strongly with accessible chromatin, consistent with its active role in accessible genome organization. *Brd2* knockout mice are embryonic lethal with severe growth and developmental defects ^58,59^. BRD2 uses the N-terminal double bromodomain to recognize acetylated histones and mediates intra- and inter-nucleosomal interactions ^51,60^.The BRD2 extra-terminal (ET) domain has been found to bind to multiple chromatin regulatory factors implicated in transcription and splicing ^61^. Except the bromodomain and the ET domain, the rest of BRD2 is highly disordered and could constitute a large floppy surface for multivalent protein-protein interactions. Chemical perturbation (JQ1 and 1,6-HD) and domain truncation experiments indicated that both protein-chromatin and protein-protein interactions are required for BRD2-mediated chromatin contacts. Indeed, by coupling loop extrusion and compartment-specific affinity interactions, the revised polymer simulation qualitatively recapitulated existing experimental observations. It would be important to comprehensively characterize the BRD2 interaction network and identify additional regulators of active compartments in future studies.

The Cohesin-dependent compartmental switch after BRD2 depletion suggest that BRD2 and its associated factors ^53,54^ could act as roadblocks at compartmental boundaries and prevent cross-boundary Cohesin extrusion. These observations echo the boundary enforcement role of BRD2-CTCF cooperation ^53^ and nucleosomal obstructions towards Cohesin movement on chromatin *in vitro* ^62^. Integrating the enhanced BRD2-chromatin interaction upon Cohesin depletion, our results suggest an interplay between BRD2 and Cohesin underlying the finely balanced formation of compartmental structures and provide a concrete molecular framework for building a coherent physical model of 3D genome folding.

The yeast BET protein homologue Bdf1 could maintain the boundary between euchromatin and heterochromatin ^63^ and the drosophila homologue was found to occupy insulator sites ^64^, suggesting an evolutionarily conserved role of BET family proteins in chromatin organization. Indeed, several studies suggest that mammalian BET family proteins could regulate higher order chromatin architecture and relevant chromatin-templated processes by interacting with distinct sets of binding partners ^65-70^. Here, we identified a competitive rather than cooperative relationship between mammalian BET family proteins BRD2 and BRD4. The depletion of BRD4 increased BRD2 chromatin binding but selectively reduced P300 chromatin occupancy and induced significantly more changes in transcriptional output than that of BRD2. These results, together with a recent report of BRD4-NIPBL pathway in chromatin looping ^70^, are consistent with a functional division of labor between BRD2 and BRD4 in mammalian genome organization and transcription, likely due to the biochemical divergency of protein domains outside the conserved bromodomain. Because development and cell fate transitions (e.g., differentiation, reprogramming) accompany extensive remodeling of the 3D genome conformation ^71-73^, it will be important to define how BET family proteins dynamically regulate genome conformation and function in these processes.

BRD4 was recently found to form ‘phase-separated’ droplets *in vitro* and puncta in living cells due to its stereotypical intrinsic disordered region (IDR) ^47^. BRD4 could regulate BRD2/3 chromatin binding through two non-exclusive mechanisms: one is through direct competitive binding to acetylated chromatin through the highly conserved double bromodomain. The other is through the formation of local high concentration protein hubs that sequester BRD2 and BRD3 away from chromatin. Recently, enhancer-associated condensates were proposed to shape genome organization ^74,75^. Furthermore, optically-induced CasDrop condensates using the BRD4-IDR were able to mechanically pull-in targeted loci and exclude non-targeted chromatin fiber ^76^. Therefore, a phase-separation mechanism has been proposed to regulate genome organization by promoting chromatin contacts, particularly for heterochromatin ^11,27,31,33^. However, our work revealed a dominant role of BRD2 in large scale accessible genome organization. Although BRD2 depends on the C-terminal domains enriched for LCD to promote chromatin contacts, whether a phase separation mechanism is invoked merits future rigorous tests and quantitative measurements ^48^.

Interestingly, although BRD2 was identified here as a functional regulator of genome compartmentalization, its acute degradation did not affect the chromatin binding of transcriptional coactivator P300 and only altered the expression of a small number of genes. On the other hand, degradation of RNA Pol II by alpha-amanitin or loss of BRD4 has a major effect on transcriptional output but does not noticeably impact the accessible genome organization. It could take time to allow the altered genome architecture to accumulate epigenetic alterations for transcription regulation ^77^. Alternatively, the molecular pathways driving large scale genome organization might separate from those involved in active transcription. Such design might allow the cell to make independent adjustments of nuclear organization (*e.g.*, shape, size, chromosome arrangement) and transcriptional output without functional entanglements under different physiological conditions (e.g., mechanical constrains, cell migration, nocturnal vision adaptation) ^78^. Transcriptional regulation is perhaps more impacted by dynamic, non-linear enhancer-promoter and promoter-promoter interactions ^43,79-82^.

Our work suggests a critical role of histone acetylation in genome topology. Histone acetylation has been shown to affect nucleosomal DNA compaction^83^. Acetylated chromatin bound by multivalent bromodomains could alter the physical properties of chromatin and induce hub formation *in vitro* ^84^. Under pathological conditions, chromatin hyperacetylation as a result of the BRD4 bromodomain-NUT fusion oncoprotein can form mega-base sub-compartments and promote intra- and inter-chromosomal chromatin interactions to drive high level oncogene expression in an aggressive form of carcinoma ^85^. And such sub-compartments are dependent on the interplay between histone acetylation and the bromodomain of fusion protein but not on transcription. Taken together, the histone acetylation and bromodomain protein interactions may have a conserved role in regulating higher-order chromatin structure in both physiological and pathological conditions. Therefore, histone acetylation binding by BRD2 and other BET proteins may present an important pharmacological target for the treatment of human cancers, infectious diseases and modulation of immune-inflammation responses ^51,86,87^. Thus, the new mechanisms we identified here regarding BET family proteins could shed new insights into novel strategies for effective disease intervention.

## Methods

### Cell culture and ESC lines generation

JM8.N4 mouse ESCs from the C57BL/6N strain and their genome edited derivatives were cultured on 0.1% gelatin coated plates without feeders at 37°C and 5% CO_2_. The ESC medium was composed of the knockout DMEM(1x) optimized for ESCs (Thermo Fisher Scientific, 10829-018), 15% ESC qualified Fetal Bovine Serum (ATCC, SCRR-30-2020), 1 mM GlutaMAX (Thermo Fisher Scientific, 35050-061), 0.1 mM MEM nonessential amino acids (Thermo Fisher Scientific, 11140-50), 0.1 mM 2-mercaptoethanol (Thermo Fisher Scientific, 21985-023),1000 units of Leukemia inhibitory factor (LIF) (Millipore) and Antibiotic-Antimycotic (Thermo Fisher Scientific, 15240-062). The JM8.N4 cells were approved by the NIH 4D Nucleome project as a Tier2 cell line.

To implement the AID system, the pLenti-EF1-osTir1-9myc-P2A-Bsd lentivirus was used to infect the low passage JM8.N4 wild type ESCs and selected with 10μg/mL Blasticidin as described previously ^24^. To generate auxin inducible degradation for Cohesin, 1.5 μg/μl SpCas9-Rad21 sgRNA-PGK-Venus construct and 3 μg/μl of RAD21 donor constructs were nucleofected into ~3×10^6 Tir1 ESCs using the Amaxa™ 4D-Nucleofector and the P3 Primary Cell 4D-Nucleofector™ X Kit following the manufacture’s protocol. ~24 hours following nucleofection, Venus positive cells were FACS sorted as a pool and grown for another ~5 days. Cells were then stained with 50nM Janelia Fluor® 549 HaloTag ligand (JF_549_, a kind gift from Luke Lavis’ lab from Janelia Research Campus) for 30min, washed 3 times with ESC medium for 15min, and subjected to another round of FACS sorting. JF_549_ positive cells were plated sparsely at 10cm tissue culture plates and grown for another ~7 days. Single colonies were picked for genotyping by designing PCR primers outside the homology arms. Bi-allelic knock-in clones were verified by Sanger Sequencing and expanded for downstream analysis. The *Rad21* sgRNAs were used as previously described ^88^. Genotyping primers for *Rad21* knock-in are: 5’-CAGGTATGCCAGCACAGTCCACA-3’, 5’- CCAGGAATACAAACCCAACCCAAA-3’.

The GFP version of RAD21-AID ESCs were similarly generated except that ESCs were sorted for GFP fluorescence ~7 days after initial sorting for Venus signal. Biallelic knock-in ESC clones were verified by PCR genotyping, Sanger Sequencing, and western blot (WB). To generate the stable SOX2-HaloTag expressing ESCs, 2μg PiggyBac_EF1_HaloTag_Sox2 was co-transfected with 1 μg PiggyBac super-transposase and selected with 500 μg/mL G418 (Thermo Fisher Scientific,10131035) for ~7 days. We further verified and selected median-level SOX2-HaloTag expressing cells by FACS sorting.

To enable orthogonal targeted degradation of BET proteins, we took advantage of the PROTAC strategy ^52^. The donor vectors for BET family genes *Brd2/Brd3/Brd4* were constructed with ~500bp homology arms flanking the FKBP^F36V^ degron sequence linked with the HaloTag (dTAG-Halo). Because BRD4 has both short and long isoforms sharing the same 5’ end ^89^, we inserted the degron to the N-terminus of *Brd4* gene, which will deplete both isoforms. The donor constructs with corresponding sgRNAs were co-nucleofected into the Rad21-mAID-eGFP lines. After ~7 days, JF_549_ positive cells were FACS sorted and single colonies were picked for genotyping by designing PCR primers outside the homology arms. Bi-allelic knock-in clones were verified by Sanger Sequencing and WB. The sgRNA sequences for BET family genes are: *Brd2* sgRNA, 5’-CGATTCAGACTCGGGCTAAG-3’; *Brd3* sgRNA, 5’-GCAGTGACTCAGAGTGAACT-3’; *Brd4* sgRNA, 5’-ACTAGCATGTCTACGGAGAG-3’.

We tested a wide range of auxin or dTAG13 concentration for RAD21 or BET protein degradation. We found that 50-500μM IAA and 100-500nM dTAG13 induced robust RAD21 or BET proteins degradation, respectively. Unless indicated, we used 100μM final concentration of IAA or 100nM dTAG13 individually or in combination throughout our experiments.

The ESC lines over-expressing the V5-SNAP tagged full length and domain truncated BRD2 were generated by transfection of 2μg corresponding PiggyBac based vectors with 1 μg PiggyBac super-transposase by Lipofectamine 3000 (Thermo Fisher Scientific, L3000015). After one week, ESCs stably integrating and expressing the BRD2 constructs were sorted for SNAP JF_552_ positive signals by flow cytometry and verified by western blot using the anti-V5 antibody.

### Chemicals and plasmids

The plant auxin analog indole-3-acetic acid (IAA) (Sigma, I3750-5G-A) was dissolved in Ethanol at a stock concentration 500mM and aliquoted to store at −20°C. 1,6 Hexanediol (Sigma, 240117-50G) were dissolved in ESC medium at final concentration 2% and treated 1.5 hours. (+) JQ-1 (MedChemExpress, HY-13030) was dissolved in DMSO at 1mM stock and aliquoted at −20°C. Alpha-amanitin (Tocris Bioscience, 4025) was dissolved at 1mg/mL stock in water, aliquoted, stored at −20°C and used at final concentration of 100μg/mL for 5 hours. dTAG13 (Tocris Bioscience, 6605) was dissolved in DMSO at 100μM stock and aliquoted at −20°C

The pLenti-EF1-osTir1-9Myc-P2A-Bsd was constructed by PCR amplifying the Oryza Sativa Tir1 (osTir1) cDNA from the pBabe Puro osTIR1-9Myc (Addgene, 80074) and inserted into the AgeI/BamHI site of the lentiCas9-Blast construct (Addgene, 52962). The donor plasmids for RAD21 were modified from previous RAD21-HaloTag donor constructs as described from ^88^ by in-frame insertion of the 71-104aa of full length auxin-inducible degron (miniAID) ^22,90^. The CRISPR/Cas9 and sgRNA constructs used for targeted RAD21 knock-in were used as previously described ^88^. The corresponding GFP version of RAD21 donor constructs were generated by replacing the HaloTag with eGFP by Gibson assembly. The PiggyBac-EF1-HaloTag-Sox2-IRES-neo and the PiggyBac super-transposase were used as previously described ^91^. The dTAG constructs was derived from pCRIS-PITChv2-dTAG-Puro (BRD4) (Addgene, 91796) and donor constructs for Brd2/3/4 were constructed by PCR amplification and Gibson assembly.

The full length (FL), N-terminus double bromodomain (BD, 1-508aa) and C-terminus region containing large segments of low complexity domains (LCDs, 447-798aa) of mouse BRD2 were PCR amplified from the RSV-Flag-mBrd2 vector (Addgene, 86614) and subcloned into a PiggyBac vector containing an N-terminus V5-SNAP tag. All plasmids used in this study are verified by Sanger Sequencing and will be available through Addgene.

### 3D ATAC-PALM imaging and quantitative analysis

We prepared the reagents for 3D ATAC-PALM experiments as described previously ^24^. Briefly, cells were plated onto #1 thickness 5mm coverslips (Warner Instruments, 64-0700) at around 70-80% confluency with proper coating one day before experiment. Coverslips were embedded with gold-nanorods as fiducial markers (generous gift from G. Shtengel, Janelia). Cells were fixed with 1% paraformaldehyde (Electron Microscopy Sciences, 15710) for 10 min at room temperature. After fixation, cells were washed three times with 1 x PBS for 5 minutes and then permeabilized with ATAC lysis buffer (10 mM Tris–Cl, pH 7.4, 10 mM NaCl, 3 mM MgCl2, 0.1% Igepal CA-630) for 10 min at room temperature. After permeabilization, the slides were washed twice in 1xPBS and put inside a humidity chamber box at 37 °C. The transposase mixture solution (1x Tagmentation buffer-10mM Tris-HCl, pH 7.6, 5mM MgCl2, 10% dimethylformamide, 100 nM Tn5-PA-JF_549_) was added to the cells and incubated for 30 min at 37 °C inside the humidity chamber. After the transposase reaction, slides were washed three times with 1 x PBS containing 0.01% SDS and 50 mM EDTA for 15 min at 55 °C before mounted onto the Lattice light-sheet microscope (LLSM) slot for 3D ATAC-PALM imaging.

The 3D ATAC-PALM data were acquired by the LLSM ^92^ at room temperature. The light sheet was generated from the interference of highly parallel beams in a square lattice and dithered to create a uniform excitation sheet. The inner and outer numerical apertures of the excitation sheet were set to be 0.44 and 0.55, respectively. A Variable-Flow Peristaltic Pump (Thermo Fisher Scientific) was used to connect a 2L reservoir with the imaging chamber with 1×PBS circulating through at a constant flow rate. Labelled cells seeded on the 5mm coverslip were placed into the imaging chamber and each image volume includes ~100-200 image frames, depending on the depth of field of view. Specifically, spontaneously activated PA-JF_549_ dye were initially pushed into the fluorescent dark state through repeated photo-bleaching by scanning the whole image view with a 2W 560 nm laser (MPB Communications Inc., Canada). Then, the samples were imaged by iteratively photo-activating each plane with very low intensity 405 nm light (<0.05 mW power at the rear aperture of the excitation objective and 6W/cm^2^ power at the sample) for 8 ms and by exciting each plane with a 2W 560 nm laser at its full power (26 mW power at the rear aperture of the excitation objective and 3466 W/cm^2^ power at the sample) for 20 ms exposure time. The specimen was illuminated when laser light went through a custom 0.65 NA excitation objective (Special Optics, Wharton, NJ) and the fluorescence generated within the specimen was collected by a detection objective (CFI Apo LWD 25×W, 1.1 NA, Nikon), filtered through a 440/521/607/700 nm BrightLine quad-band bandpass filter (Semrock) and N-BK7 Mounted Plano-Convex Round cylindrical lens (f = 1000 mm, Ø 1", Thorlabs), and eventually recorded by an ORCA-Flash 4.0 sCMOS camera (Hamamatsu). The cells were imaged under sample scanning mode and the dithered light sheet at 500 nm step size, thereby capturing a volume of ~25 μm × 51 μm × (27~54) μm, considering 32.8° angle between the excitation direction and the stage moving plane.

To precisely analyze the 3D ATAC-PALM data, we used gold-nanorods fiducials embedded within the coverslips for drift correction as previously described ^93^. ATAC-PALM Images were taken to construct a 3D volume when the sample was moving along the “s” axis. Individual volumes per acquisition were automatically stored as Tiff stacks, which were then analyzed by in-house scripts written in MATLAB. The cylindrical lens introduced astigmatism in the detection path and recorded each isolated single molecule with its ellipticity, thereby encoding the 3D position of each molecule relative to the microscope focal plane. All processing was performed by converting all dimensions to units of xy pixels, which were 100 nm × 100 nm after transformation due to the magnification of the detection objective and tube lens. We estimated the localization precision by calculating the standard deviation of all the localizations coordinates (x, y and z) after the gold-nanorods fiducial correction. The localization precision is 26±3 nm and 53±5 nm for xy and z, respectively. For more details of quantitative analysis of 3D ATAC-PALM please refer to our previous publication ^24^.

### 3D Imaging of SOX2 enhancer clusters in live cells

ESCs stably expressing HaloTag-SOX2 in the RAD21-GFP-AID genetic background were plated onto #1 5mm round coverslips (Warner Instruments, 64-0700, CS-5R) pre-coated with Corning Cell-Tak matrix (Corning, 354240). Cells were stained with HaloTag ligand JF_549_ at a final concentration of 10 nM for 15 mins, washed in PBS twice before being mounted onto the sample holder. The imaging medium was prepared by supplementing FluoroBrite DMEM Medium (Thermo Fisher Scientific) with 15% ESC Qualified Bovine Serum, 1 mM Glutamax, 0.1 mM nonessential amino acids, 1 mM sodium pyruvate, 0.1 mM 2-mercaptoethanol, and 1000 units of LIF. Before experiments, the LLSM was calibrated with imaging medium at 37^o^C overnight. During experiments, the imaging chamber was filled with 8 mL imaging medium containing 40pM HaloTag ligand JF_549_. All live cell samples were imaged by iteratively exciting each plane with a 560 nm laser (~10 mW power at the rear aperture of the excitation objective) at 50 ms exposure time. The sample cells were imaged around 200 frames per sample volume by sample scanning mode with the dithered light sheet of 500 nm step size, thereby capturing a volume of ~25 μm × 51 μm × 54 μm.

3D localization (x, y, z) of detected single molecules was further analyzed as previously described ^37^. The PSF model can be described as below:

$$I(x,y,z)=A_{0}\cdot e^{-\left(\frac{(x-x_{0}{)}^{2}}{2\sigma_{x}^{\;\;2}}+\frac{(y-y_{0}{)}^{2}}{2\sigma_{y}^{\;\;2}}+\frac{(z-z_{0}{)}^{2}}{2\sigma_{z}^{\;\;2}}\right)}+B$$

Where *A_0_* is the signal amplitude and *B* is the background signal value.

Image registration and drift correction were similarly performed for the ATAC-PALM methods. The centroid displacement of total localization events from every 40 time points (160 s) was calculated and the resulting transformation matrix over time was applied to the data accordingly. Significant drifted datasets were discarded in the following tracking analysis.

We previously demonstrated that residence time was one valid strategy to distinguish TF SOX2 specific binding sites from non-specific binding sites by live cell imaging ^37,91^. Therefore, we used a residence time cut off (4 seconds) as previously demonstrated for SOX2 ^37^ to filter out non-specific binding events and reconstruct the SOX2 enhancer cluster for auto-correlation analysis in live cells.

### DBSCAN analysis

The density-based clustering algorithm DBSCAN (Density-Based Spatial Clustering of Applications with Noise) was adopted to map and visualize individual local ACDs (core DBSCAN MATLAB code from http://yarpiz.com/255/ypml110-dbscan-clustering) as we previously described ^24^. The algorithm first finds neighboring data points within a sphere of radius *r* and adds them into same group. In parallel, a predefined threshold of minimal points (*minPts*) was used by the algorithm to justify whether any counted group is a cluster. If the number of points within a group is less than the threshold *minPts*, the data point is classified as noise. As a negative control, we generated uniformly sampled data sets with the same localization density as our ATAC-PALM localizations. We then implemented DBSCAN analysis by using 150 nm as the searching radius (*r*) (peak radius from the Ripley’s H function analysis) and empirically setting *minPts* as 10. To reconstruct the *iso*-surface for each identified ACD, the convex hull which contains the ACD data points was calculated and visualized by using MATLAB. The volume of the convex hull was computed, and the normalized cluster radius (calculated from a sphere with equal volume) was estimated and shown in violin plot.

### Oligopaint FISH experiment and analysis

The Oligopaint FISH probe libraries were constructed as described previously ^94^. A Tier 15 ssDNA oligo pool was ordered and synthesized from Twist Bioscience (San Francisco, CA). Each oligo consists of a 32 nucleotide (nt) homology to the mm9 genome assemble discovered by the OligoArray2.0 with the following parameters -n 22 -D 1000 -l 32 -L 32 -g 52 -t 75 -T 85 -s 60 -x 60 -p 35 -P 80-m "GGGGG;CCCCC; TTTTT;AAAAA" run from the algorithm developed from the laboratory of Dr.Ting Wu (https://oligopaints.hms.harvard.edu/). Each library subpool consists of a unique sets of primer pairs for orthogonal PCR amplification and a 20 nt T7 promoter sequence for *in vitro* transcription and a 20 nt region for reverse transcription. Individual Oligopaint probes were generated by PCR amplification, *in vitro* transcription, and reverse transcription, in which ssDNA oligos conjugated with ATTO565 and ATTO647 fluorophores were introduced during the reverse transcription step as described previously ^95,96^. The Oligopaint covered genomic region (mm9) used in this study are listed below:

**Table:** 

Domain Name	Genome coordinates (mm9)	Length (kb)	Probe number
Domain R1	Chr4: 53067000-53700000	633	8948
Domain R2	Chr4: 54925000-55568000	643	9324
Domain R3	Chr4: 56810000-57930000	1120	16553
Domain R4	Chr6: 120150000-121400000	1250	17717
Domain R5	Chr6: 122160000-122800000	640	8007
Domain R6	Chr6: 124210000-125200000	990	11763
Locus_Chr4_Red	Ch4: 55459131-55474138	15	204
Locus_Chr4_Green	Ch4:57319865-57334978	15	243
Locus_Chr6_Red	Chr6:120295072-120306069	11	197
Locus_Chr6_Green	Chr6: 122591480-122609134	18	198

**Table:** 

Domain Name	Forward primer (5’-3’)	Reveres primer (5’-3’)	RT oligo (5’-3’)
Domain R1	GCGGGACGTAAGGGCAACCG	GCGTTGCGGTGCGATCTTCT	CCTATCCCCTGTGTGCCTTG
Domain R2	TTGGGTCCGGTTGTGATCCG	GCGATGCCCGGGTAACACAA	AATTCGGCAGACCCGAATGC
Domain R3	TGATAACCCACGCACGGCTG	GACCCGGGCCACTAACACGA	AATTCGGCAGACCCGAATGC
Domain R4	CACGGCAACCCTCAGAACGG	CAGTTCGGTGGGACCGGGTT	CCTATCCCCTGTGTGCCTTG
Domain R5	TTGGACGGCGCGCGTAAGAC	GGATTGCGCTCATGCCGTCT	CCTATCCCCTGTGTGCCTTG
Domain R6	ACGTCCATGCAAGGAACGGG	CACGTGACGTCGGTTGGACG	AATTCGGCAGACCCGAATGC
Locus_Chr4_Red	ATTCATATGCGCTCCGGCGG	GAGCCCGCTGATACACGCGC	AATTCGGCAGACCCGAATGC
Locus_Chr4_Green	GACGTTTCATCGGACGCCCG	CCGGCTCGGGAGTCGACAAT	CCTATCCCCTGTGTGCCTTG
Locus_Chr6_Red	GGGAGTAGGGTCCTTTGTGTG	TTCTCTAGAACGATCCAGCGA	CCTATCCCCTGTGTGCCTTG
Locus_Chr6_Green	TGTCATGGTGGATCGGCAGC	GATGCCGACGCGAACACCAT	AATTCGGCAGACCCGAATGC

The oligo sequences for conjugating fluorophores during reverse transcription are:

/5ATTO565N/ or /5ATTO647N/AATTCGGCAGACCCGAATGC

/5ATTO565N/ or /5ATTO647N/CCTATCCCCTGTGTGCCTTG

For 3D DNA FISH on ESCs, #1.5 round glass coverslips (Electron Microscopy Sciences) were pre-rinsed with anhydrous ethanol for 5min, air dried, and coated with 0.1% gelatin or equivalent for at least 2 hours. Fully dissociated ESCs were seeded onto the coverslips and recovered for at least 6 hours before experiments. To mitigate potential cell cycle impacts for DNA FISH experiments after Cohesin depletion ^97^, we reduced the auxin treatment time from 6 hours to 3 hours for Oligopaint experiments measuring chromatin compaction. 3-hour auxin treatment still robustly depleted RAD21 (Extended Data Fig.1a-b) but had negligible effect on cell cycle progression (data not shown). 1μM JQ1 treatment for 12 hours or 2% 1,6-HD treatment for 1.5 hours also did not cause detectable cell cycle defects (data not shown). Cells were fixed with 4% (v/v) methanol free paraformaldehyde (Electron Microscopy Sciences, 15710) diluted in 1xPBS at room temperature for 10min. Then cells were washed twice with 1xPBS and permeabilized in 0.5% Triton-X100 in 1xPBS for 30min. After two times wash in 1xPBS, cells were treated with 0.1M HCl for 5min, followed by three washes with 2xSSC and 30 min incubation in 2x SSC + 0.1% Tween20 (2xSSCT) + 50% (v/v) formamide (EMD Millipore, S4117). For each sample, we prepare 25μl hybridization mixture containing 2xSSCT+ 50% formamide +10% Dextran sulfate (EMD Millipore, S4030) supplemented with 0.5μl 10mg/mL RNaseA (Thermo Fisher Scientific, 12091-021) +0.5μl 10mg/mL salmon sperm DNA (Thermo Fisher Scientific, 15632011) and 20pmol probes with distinct fluorophores. The probe mixture was thoroughly mixed by vortexing, and briefly microcentrifuged. The hybridization mix was transferred directly onto the coverslip which was inverted facing a clean slide. The coverslip was sealed onto the slide by adding a layer of rubber cement (Fixo gum, Marabu) around the edges. Each slide was denatured at 78°C for 3 min followed by transferring to a humidified hybridization chamber and incubated at 42°C for 16 hours in a heated incubator. After hybridization, samples were washed 2x for 15 minutes in pre-warmed 2xSSCT at 60 °C and then were further incubated in 2xSSCT for 10min at RT, in 0.2xSSC for 10min at RT, in 1xPBS for 2x5min with DNA counterstaining with DAPI. Then coverslips were mounted on slides with Prolong Diamond Antifade Mountant (Thermo Fisher Scientific, P36961) for imaging acquisition.

3D DNA FISH images were acquired on the ZEISS LSM 880 Inverted Confocal microscope attached with a Airyscan 32 GaAsP (gallium arsenide phosphide)-PMT area detector ^98^. Before imaging, the beam position was calibrated centering on the 32-detector array. Images were taken under the Airyscan Super-resolution mode with a Plan Apochromat 63x/NA1.40 oil objective in a lens immersion medium having a refractive index 1.515. We used 405nm (Excitation wavelength) and 460nm (Emission wavelength) for the DAPI channel, 561nm (Excitation wavelength) and 579nm (Emission wavelength) for the ATTO565 channel and 633nm (Excitation wavelength) and 654nm (Emission wavelength) for the ATTO647 channel. Z-stacks were acquired under Super-resolution mode for the optimal z sectioning thickness around 190nm. The Airyscan super-resolution technology used a very small pinhole (0.2AU) at each of its 32 detector elements to increase SNR ~4-8 fold and enables ~1.7-fold improvement of resolution upon linear deconvolution in both lateral (xy) and axial (z) directions. After image acquisition, Airyscan image was post-processed and reconstructed at the auto processing strength using the provided algorithm from ZEISS LSM880 platform.

3D DNA FISH analysis was performed in Imaris 9.1 installed in Windows 10 X64 OS with the GeForce GTX 760/PCIe/SSE2 (version 4.5.0 NVIDIA 369.09). We applied a background subtraction filter to the 3D Airyscan processed images before the downstream analysis. To characterize the 3D DNA-FISH domain, we employed the synthetic model—Surfaces object from Imaris and applied a Gaussian filter (σ = 1 voxel in xy) before the downstream 3D segmentation and quantification. We manually inspected the images and removed the DNA-FISH signal for downstream analysis meeting the following criteria: (a) unexpected, non-specific signal outside the DAPI-stained nuclei, (b) FISH signal in highly condensed or apparently double-sized nuclei (mitotic or G2 stage), (c) cropped signal at the edge of the images, (d) very faint signal. The 3D volume of the DNA FISH defined domain is estimated by the number of voxels within the detected objects with the voxel size (48.9nmX48.9nmX199nm). To measure the 3D distance between foci-pairs, we localized the voxels corresponding to the local maximum of identified DNA FISH signal using the Imaris Spots function module and calculated the Euclidean distance accordingly. Chromatic aberrations in the imaging system presented as a systematic shift among the visible wavelengths within the field of view. This distance shift was quantified using 0.5 μm multi-spectral beads under the same acquisition settings. In lieu of performing a channel alignment which does not correct for sub-pixel length shifts, the measured distance offset between the relevant fluorescent channels was appropriately subtracted from all downstream 3D loci pair-distance measurements.

### ATAC-seq libraries preparation and genomic analysis

ATAC-seq libraries were made according to the published protocols ^99,100^ using the Nextera DNA Library Preparation Kit (Illumina, FC-121-1030) or home-made Tn5 transposon as described in ^101^. We also supplemented 0.05ng drosophila genomic DNA purified from S2 cells right before adding the tagmentation mixture to improve comparison between samples. Multiplexed ATAC-seq libraries (barcodes were adapted from ^99^ ) were sequenced on Illumina HiSeq 2500 or NextSeq 500/550 high and/or mid throughput 150 cycles at Janelia quantitative Genomics Core, with a run configuration of 75 bp paired-end sequencing, either as single indexed or dual indexed runs. All samples were quantitated on Roche 480 lightcycler using FAST qPCR program and normalized and pooled at 2nM. Libraries were loaded at varied final concentrations across HiSeq (10pM) and NextSeq (1.5pM to 1.9pM). Illumina’s Bcl2tofastq2 v2.17 was used to convert BcL files to fastq files and to demultiplex the samples.

To analyze ATAC-seq libraries, pair-end reads were first adapter removed by Cutadapt and mapped to mm10 genome build using Bowtie2 with the following parameters: --no-discordant --no-mixed --phred33 -X2000 –threads32. Reads mapped to mitochondria/uncharacterized chromosomes (chrM/chrUn/random) and PCR duplicates were removed by samtools. The pair-end reads from the drosophila genomic DNA was mapped to dm6 genome build. To compare the coverage among ATAC-seq libraries, sequencing reads were normalized to 1×sequence depth defined by total number of mapped reads × fragment length / effective genome size (2,150,570,000) or by the spike-in drosophila genomic DNA reads. ATAC-seq peaks were called using the MACS2 callpeak function using the –f BAMPE parameter. Fragment length distribution, TSS enrichment, scatter plots were analyzed as previously described ^102^. Raw sequencing data were deposited to NCBI GEO with the accession number of GSE163729.

CTCF and RAD21 ChIP-seq data sets from wild type ESCs were obtained from ^88^ under GSE number GSE90994. The Transcription start site (TSS) annotation was downloaded from the UCSC Table Browser mouse mm10 build (GRCm38/mm10, Dec.2011) NCBI RefSeq genes and the promoters were defined as −400bp to +100bp relative to the TSS. ESC enhancer coordinates were retrieved from the H3K27ac ChIP-seq peak regions as described previously ^103^. RNA-seq data to query BET family gene expression levels were derived from GSM723776 and GSM723775.

### ChIP-seq and CUT&Tag

ChIP experiments were performed as described previously ^104^. Briefly, cells were cross-linked for 10 min at room temperature with 1% methanol-free paraformaldehyde in 1xPBS and quenched with 0.125 M glycine. Cells were scraped and resuspended in cold cell lysis buffer (5 mM PIPES at pH 8.0, 85 mM KCl, 0.5% NP-40) and incubated for 10 min on ice. After centrifuging, nuclear pellets were resuspended in at least 6 vol of sonication buffer (50 mM Tris-HCl at pH 8.1, 10 mM EDTA, 0.1% SDS), incubated for 10 min on ice, and sonicated using the Covaris S220 sonicator to obtain an average fragment length of ~500 bp examined by electrophoresis. Sonicated chromatin was diluted in RIPA buffer, aliquoted, and incubated with Protein G Dynabeads that were prebound with individual antibodies for at least 1 h at room temperature. Immunoprecipitation was performed overnight at 4°C with 2–4 μg of antibodies. Ten percent of the chromatin was saved as input. Immunoprecipitated and input DNA was introduced with sequencing adaptors as described previously ^105^, washed extensively with RIPA buffer (low salt and high salt) and LiCl buffer, digested with proteinase K and RNase A, reverse-cross-linked at 60°C overnight followed by purification with the AmpureX beads. We also added the spike-in chromatin (active motif, 53083) and spike-in antibody (active motif, 61686) following the manufacturer’s instructions. The antibodies used for ChIP-seq assay are IgG (Novus Biologicals, NB810-56910), BRD2(Bethyl laboratories, A302-583A), BRD3(Active Motif, 61489), BRD4(abcam, ab128874), CTCF (Millipore, 07729), RAD21 (abcam, 154769), p300 (santa cruze, sc48343x).

Pair-end reads from ChIP-seq libraries were first adapter removed by Cutadapt and mapped to mm10 genome build using Bowtie2 with the following parameters: --no-discordant --no-mixed --phred33 -X2000 –threads32. Reads mapped to mitochondria/uncharacterized chromosomes (chrM/chrUn/random) and PCR duplicates were removed by samtools. The pair-end reads from the drosophila genomic DNA was mapped to dm6 genome build. To compare the coverage among ChIP-seq libraries, sequencing reads were normalized to 1×sequence depth defined by total number of mapped reads × fragment length / effective genome size (2,150,570,000) or by the spike-in drosophila chromatin reads whenever possible. ChIP-seq peaks were called using the MACS2 callpeak function using the –f BAMPE parameter with --broad function for broad peak calling for BET proteins.

BRD2 CUT&Tag was performed as previously described^106^ by using the primary anti-BRD2 antibody from Bethyl Laboratory (A302-583A) or Cell Signaling Technology (5848S) . pAG-Tn5 was ordered from EpiCypher (15-1017). Due to the high sensitivity and low background, we used SEACR package for peak calling with default parameters considering normalization^107^.

### In situ Hi-C and Micro-C

In situ Hi-C experiment was performed in non-treated control and RAD21 depleted ESCs after 6 hour auxin treatment as described previously ^108^. Using Juicer software ^109^, .hic files were generated, and normalized ligation frequency matrices were obtained with the dump command. Interaction matrices were subsequently visualized by Juicebox software. The final bin resolution of Hi-C map is 5kb. Instead of calling Hi-C domain, we merged ATAC-seq peaks and called ATAC-rich regions for analysis. The contact probability profile on distance and intra-/inter-domain interactions were calculated as described ^108^. The inter-ATAC domain interaction was calculated only between adjacent domains but not from all possible combinations.

Micro-C experiments were performed as previously described ^43^. Briefly, 5 million ESC cells were resuspended by trypsin, fixed by freshly made 1% formaldehyde at room temperature for 10 min and quenched by adding Tris buffer (pH = 7.5) to the final 0.75M at room temperature. Fixed cells were washed twice with 1X PBS and then were subjected to the second crosslinking reaction by 3 mM disuccinimidyl glutarate (DSG) (Thermo Fisher Scientific, 20593) for 45 min at room temperature. The DSG solution was freshly made at a 300mM concentration in DMSO and diluted to 3 mM in 1x PBS before use. The crosslinking reaction was quenched by 0.75 M Tris buffer and washed twice with 1x PBS. Crosslinked cells were snap-frozen in liquid nitrogen and stored at −80°C.

We performed chromatin fragmentation by the micrococcal nuclease (MNase) and optimize the MNase digestion level to have about 80 to 90% of mononucleosomes and 10 to 20% of di-nucleosomes. We typically use ~20 units MNase for 1 million JM8.N4 ESCs. Intact nuclei were extracted by treating cells with Micro-C Buffer #1 (50 mM NaCl, 10 mM Tris-HCl pH = 7.5, 5 mM MgCl_2_, 1M CaCl_2_, 0.2% NP-40, 1x Protease Inhibitor Cocktail) for 20 min on ice. Chromatin was digested with a pre-titrated MNase concentration (Worthington Biochem , LS004798) at 37°C for 10 min. MNase digestion was stopped by adding 4 mM EGTA and completely inactivated by incubating at 65°C for 10 min. Digested chromatin was washed twice with ice-cold Micro-C Buffer #2 (50 mM NaCl, 10 mM Tris-HCl pH = 7.5, 10 mM MgCl_2_).

MNase digested chromatin was subjected to multiple steps of biochemical enzyme reactions to generate compatible ends for ligation of adapters for sequencing. Chromatin was incubated with T4 PNK (New England BioLabs, M0201) in Micro-C end-repair buffer (50 mM NaCl, 10 mM Tris-HCl pH = 7.5, 10 mM MgCl_2_, 100 ug/mL BSA, 2 mM ATP, 5 mM DTT) at 37°C for 15 mi. We then incubate chromatin with Klenow Fragment in the Micro-C end-repair buffer with no dNTPs at 37°C for 15 min. The blunting and labeling reaction was triggered upon adding biotin-dATP (Jena Bioscience, NU-835-BIO14), biotin-dCTP (Jena Bioscience, NU-809-BIOX), dGTP, and dTTP to a final concentration of 66 mM each at room temperature for 45 min. The reaction was inactivated by adding 30 mM EDTA and incubating at 65°C for 20 min. Biotin-labeled chromatin was washed once by ice-cold Micro-C Buffer #3 (50 mM Tris-HCl pH = 7.5, 10 mM MgCl_2_). The crosslinked nucleosomes were then ligated by T4 DNA Ligase (New England BioLabs, M0202) in 500 μL solution at room temperature for at least 2 h, followed by exonuclease III (New England BioLabs, M0206) treatment to remove biotin-dNTPs on un-ligated ends by incubating ligated chromatin at 37°C for at least 15 min.

To specifically extract the ligated di-nucleosomal DNA, the deproteinized chromatin was purified and separated on a low-melting agarose gel (Lonza, 50081). A band at the size of 250 to 400 bp corresponding to the ligated dimers was gel-extracted for library preparation. The purified DNA with biotin-dNTPs was captured by Dynabeads® MyOne Streptavidin C1 (Thermo Fisher Scientific, 65001). Standard Illumina library preparation protocol including end-repair, A-tailing, and adaptor ligation was performed on beads with the NEBnext Ultra II kit (New England BioLabs, E7645). An optimal PCR cycle for final library amplification was determined by quantification PCR (KAPA Biosystems, KK4602), typically between 5–10 cycles for the input cell number from 5M. The sequencing library was amplified by Kapa HiFi PCR enzyme (KAPA Biosystems, KK2601) with the lowest possible cycles to reduce PCR duplicates. More details about Micro-C analysis can be found in previous published study ^43^.

### Micro-C data analysis

#### Preprocessing Micro-C data

Valid Micro-C contact read pairs were obtained from the HiC-Pro analysis pipeline ^110^. The detailed description and code can be found at https://github.com/nservant/HiC-Pro. In brief, each fastq file was mapped to the mus musculus (mm10) genome separately using Bowtie 2.3.0 ^111^ with ‘very-sensitive-local’ mode. Aligned reads were paired by the read name. Pairs with multiple hits, low MAPQ, singleton, dangling end, self-circle, and PCR duplicates were discarded. Contacts shorter than 200bp (unligated mono-nucleosomes) were also removed. Output files containing all valid pairs were used in downstream analyses. We recommend checking the statistics for initial quality control before moving forward to the downstream analysis: 1) bowtie mapping rate; 2) reads pairing percentage; 3) ratio of sequencing artifacts; 4) ratio of cis/trans contacts; 5) unligated monomer percentage. If any of the above results are not in the optimal range as reported in the previous studies, one might consider adjusting mapping and filtering parameters or further optimize the Micro-C experiment.

Valid Micro-C contacts were assigned into the bin with the corresponding coordinates. The bin file was pre-generated from the mm10 genome by the 1kb window. The binned matrix can be stored in HDF5 format as COOL file format by using COOLER package (https://github.com/mirnylab/cooler) ^112^ or HIC file format by using JUICER package (https://github.com/aidenlab/juicer) ^109^. COOL and HIC are the standard 4DN formats that are compatible with many downstream analysis ecosystems (https://www.4dnucleome.org/). Contact matrices were then normalized by using iterative correction (IC) in COOL files ^113^ or Knight-Ruiz (KR) ^114^ in HIC files. Regions with low mappability and high noise were blocked before matrix normalization. We expect that matrix balancing normalization corrects systematic biases such as nucleosome occupancy, sequence uniqueness, GC content, or crosslinking effects as reported in previous studies ^10,113^.

To visualize the Micro-C contact maps, we generated a compilation of COOL file with multiple resolutions (1kb to 10Mb) that can be browsed on the Higlass 3D genome server (http://higlass.io) ^115^. Alternatively, data in the HIC format can be browsed with the Juicebox browser. In this study, all snapshots of Micro-C or Hi-C contact maps, including normalized obs, obs/exp, and Pearson’s correlation matrices, were generated from HiGlass or Juicebox.

#### Compartment analysis

Chromosome compartments were identified by Principal Component Analysis (PCA) of the contact matrix at 100-kb or 200-kb resolution. The eigenvectors of the first component typically represent the compartment profile in Hi-C data ^6^ as positive values are the A compartment (gene-rich or active chromatin) and negative values are the B compartment (gene-poor or inactive chromatin). The directionality of eigenvectors was further corrected by the GC ratio or active histone marks. Histograms of the values of eigenvectors were used to show the compartment switches in various depletion conditions.

The saddle plot represents the rearrangement and aggregation of genome-wide distance-normalized contact matrix with the order of increasing eigenvector values. The upper-left and bottom-right represent the contact frequency between B-B and A-A compartments and the upper-right and bottom-left show the frequency of intercompartment A-B or B-A interactions. The interactions between strong compartment A were extracted from the saddle matrix.

#### Pileup analysis for loop or domain

To examine whether each depletion condition has an impact on the loop intensity, we assessed the genome-wide loop intensity by the aggregate peak analysis (APA) from JUICER ^109^ or coolpuppy ^116^ packages. The list used for peak aggregation in mouse ESCs was from Hsieh et al ^43^. In brief, loops were centered and piled up on a 50kb x 50kb matrix with 1kb resolution data. The pileup matrix was then normalized with matrix balance. Short-range loops were excluded or normalized by a random shift to avoid distance decay effects. The ratio of loop enrichment was calculated by dividing normalized center contacts in a searching window by the normalized corner submatrices.

For aggregate domain analysis (ADA), each domain was rescaled to a pseudo-size by N_i,j_=((C_i_-D_start_)/(D_end_-D_start_), (C_j_-D_start_)/D_end_-D_start_)), where C_i,j_ is a pair of contact loci within domain D that is flanked by D_start_ and D_end_, and N_i,j_ is a pair of the rescaled coordinates. The rescaled domains were aggregated at the center of the plot with ICE or distance normalization. The list of domains was acquired from Hsieh et al ^43^.

### RNA-seq

BRD2-dTAG or BRD4-dTAG cells were dTAG13 depleted for 6 hours with each condition in 3 biological replicates. Total RNA was isolated according to the manufacturer’s instruction, quantified by Nanodrop (Thermo Fisher Scientific) and diluted to 1 ng/μl in nuclease-free water. A total of 1 ng of RNA was added to 2.5 μl of cell lysis buffer (nuclease-free water with 0.2 % v/v Triton X-100 and 0.1 U/μl RNase inhibitor), subjected to cDNA synthesis and amplification as described before ^117^. Libraries were prepared using a modified Nextera XT DNA protocol (Illumina, San Diego, CA) where 5 μM P5NEXTPT5 was substituted for the i5 primers in the kit. Libraries were quantified by qPCR (Roche), normalized to 2 nM, then pooled and sequenced on a NextSeq550 flowcell with 25 bases in read 1, 8 bases in the i7 index read, and 50 bases in read 2. The control library phiX (Illumina) was spiked in at a final concentration of 15% to improve color balance in read 1.

Smrtscrb2 analysis pipeline: Custom python scripts were used to extract Barcode and UMI sequences from read 1. The correction of barcode error was achieved using starcode v1.1 ^118^ with the following additional parameters: “-d 1 -q --print_clusters”. Read 2 sequences were renamed using the error-corrected barcode from starcode and UMI sequences from read 1, and were aligned to the Homo sapiens GRCh38.p12 genome assembly and annotation from Ensembl (ensembl.org) using STAR ^119^ with the following additional parameters: “--alignIntronMax 200000 --outSAMattributes All --outSAMunmapped Within --outSAMtype BAM Unsorted”. The validity of an alignment was defined by the unique alignment to an exon feature on the correct strand. Gene-level counts were created using valid alignments with at least 50% of the read aligned to an exon feature, using a custom python script to collapse UMIs by gene. Differential gene expression was analyzed by DESeq2 ^120^. By setting p value 0.05 as a significance test cut off, we identified 133 genes significantly changed after 6-hour BRD2 depletion whereas 1579 genes were significantly changed for 6-hour BRD4 depletion. All custom scripts are available by request.

### Co-immunoprecipitation (Co-IP)

Co-IP was performed as describe previously ^88,104^. Mouse ESCs grown on two 15cm tissue culture plates were scraped off the plate with ice-cold 1xPBS and centrifuged, and the cell pellet was resuspended in ice-cold cell lysis buffer (5 mM PIPES at pH 8.0, 85 mM KCl, 0.5% NP-40, protease inhibitors) and incubated for 10 min on ice. Nuclei were pelleted by centrifuge at 4°C and resuspended in 1mL of 0.1M co-IP buffer (0.1 M NaCl, 25 mM HEPES, 1 mM MgCl_2_, 0.2 mM EDTA, 0.5% NP-40, protease inhibitors) followed by a brief sonication at 4°C. Aliquot the nuclear protein lysate equally into two tubes, one for untreated (UT) control and the other for Benzonase treatment (1μl per tube) rocked at 4°C for 2 hours. Then bring the salt concentration back to 0.3M with 5M NaCl, rock at 4°C for 1 hour, centrifuge at maximal speed at 4°C, and then transfer the supernatant to a new tube. The lysate was pre-cleared with the Pierce Protein G magnetic beads (Thermo Fisher Scientific, 88847) and then incubated with 4 μg of IgGs or specific BRD2 (Bethyl laboratories, A302-583A) or CTCF (abcam, ab70303) antibodies rocked overnight in a cold room. Fifty microliters of precleared lysate were kept overnight at 4°C as input. The next day, ~50 μl of Protein G magnetic beads in co-IP buffer supplemented with 0.5% BSA was added to the samples and incubated for 2 h in a cold room. After extensive washes in 0.3M salt co-IP buffer, proteins were eluted from the beads by boiling for 5 min in 1× SDS loading buffer and analyzed by WB.

### Western Blots

ESCs were lysed in 1xSDS sampling buffer (200mM Tris HCl pH7, 10% glycerol, 2% SDS, 4% beta-mercaptoethanol, 400mM DTT, 0.4% bromophenol blue) preheated at 95°C. Lysates were further sonicated and denatured at 95°C for 5min. Proteins from each sample was resolved by SDS-PAGE using Mini-PROTEAN® TGX™ Precast Gels (Bio-Rad). Primary antibodies used include: CTCF (Millipore, 07729), RAD21 (abcam, 154769), OCT4 (Santa Cruz, sc-5279), SOX2 (Millipore, AB5603), BRD2(Bethyl laboratories, A302-583A), BRD3(Active Motif, 61489), BRD4(Abcam, ab128874 for both short and long isoforms ) or BRD4 (Active Motif, 39010, for long isoform only). We used HRP conjugated secondary antibodies (Pierce) at a dilution of 1:3000. Western blot was exposed to Western Lightning Plus-ECL (PerkinElmer) and imaged in a ChemiDoc MP (Bio-Rad) detection system.

### Immunofluorescence, imaging and analysis

ESCs were grown in Nunc Lab-Tek chamber slides, washed with PBS, pre-extracted as previously described ^121^, and fixed with 4% freshly made formaldehyde for 10 minutes at room temperature (RT). Cells were permeabilized with 0.5% Triton X-100 in PBS for 10 minutes at RT and blocked with 2% BSA in 0.1% Triton-X 100 in PBS for 30min. Different primary antibodies were diluted in blocking buffer and applied to cells overnight in the cold room with gentle rotation. Primary antibodies used in the current study are: anti-HA (abcam, ab18181, 1:200), anti-BRD2 (Bethyl Laboratories, A302-583A, 1:200), anti-BRD4 (abcam, ab128874, 1:200), anti-RNA Polymerase II RPB1 antibody (BioLegend, H14, 920304, 1:500), anti-OCT4 (Santa Cruz, C-10, 1:250). After three washes. secondary antibody (1:1000) conjugated with appropriate fluorophores were applied to the cells and incubated at RT for 1hour.

Immunofluorescence and HaloTag JF_549_ imaging for RAD21 or BET depletion was performed under the Zeiss LSM880 system with Airyscan. We used the default excitation lines 405/488/561/633 nm lasers and the multi-Alkali and 32-channel GaAsP PMT detectors. Multiple z-stack images at the optimal super-resolution mode setting were acquired. Colocalization analysis was performed in ImageJ/Fiji2 as described previously ^122^.

### Cell cycle and cell proliferation analysis

RAD21 depleted ESCs were assayed for DNA replication by the Click-iT® EdU Alexa Fluor® 488 Flow Cytometry Assay Kit (Thermo Fisher Scientific, C-10420) according to the manufacture’s protocols. Prior to Flow Cytometry analysis, DNA was stained with 1μg/mL FxCycle™ Violet stain (Thermo Fisher Scientific, F10347) at room temperature for 30min. DNA synthesis was measured by incorporating 5-ethynyl-2’-deoxyuridine (EdU) coupled with an alkyne group, followed by the click reaction with Alexa Fluor 488 dye coupled with the picolyl azide group. Flow Cytometry was performed on the CytoFLEX S system from Beckman with 50,000 cells recorded and analyzed by FlowJo.

Cell cycle analysis was performed by propidium iodide staining following the protocols from the propidium iodide (PI) flow cytometry kit (abcam, ab139418). Briefly, cells were trypsinized into single cell suspension and fixed with 67% ice-cold ethanol in 1xPBS overnight at 4°C. The next day, the cells were rehydrated with 1xPBS and then stained with propidium iodide (final 50μg/mL) and RNaseA (final 500 unit/mL) for 30min at 37°C before flow cytometry analysis. All samples were acquired on a Beckman Coulter CytoFLEX S with 4 lasers (405 nm, 488 nm, 561 nm, 638 nm) (BA24075, Beckman Coulter, Brea, CA), and operated using CytExpert Software v2.3 (Beckman Coulter, Brea, CA), using 488 nm FSC-H (488-FSC-H) as the threshold parameter (threshold Automatic setting). Detector gain for fluorescence and FSC/SSC detection were optimized using control cells without treatment. The gains for the respective detectors associated with the following spectral filters: 488-FSC, 488-SSC, 561-585/42 and 561- 610/20 were 39, 15, 28, and 10 a.u. respectively. SSC-A versus FSC-W was used for initial gating of singlet cells followed by SSC-A versus FSC-A to further define cellular events. An event count versus 561-610/20 histogram plot was used to determine the percentage of cells in G1, S-phase or G2 of the cell cycle. All samples were acquired for 5 minutes at a sampling rate of 30 μl/min or up to 10,000 cells. Flowjo v.10.7.1 (Flowjo, LLC, Ashland, OR) was used for analysis of flow cytometry data.

### Chromatin fractionation assay

Salt fractionation of mouse ESC chromatin was described as previously reported ^123,124^. The same starting amount of untreated control and Cohesin depleted (6 hours auxin treatment) ESCs from 1X150mm plate was used for nuclei isolation and increasing salt extraction (80mM, 150mM, 300mM, 600mM NaCl). Equal amount of material from each fraction in untreated control and Cohesin depleted condition was analyzed by WB. The enrichment of GAPDH only in the total cell fraction and histone H4 only in the high salt fraction (300mM) indicates the successful chromatin fractionation.

### Single particle tracking experiment and analysis

Single particle tracking (SPT) experiments were primarily carried out as described in ^91,104^. Briefly, cells were seeded on 25mm #1.5 coverglass pre-cleaned with KOH and ethanol and coated with Matrigel according to manufacturer’s instruction. All live cell imaging experiments were conducted using an ESC imaging medium composed of FluoroBrite DMEM (Thermo Fisher Scientific, A1896701) plus 15% FBS, 1xGlutaMax, 1xNEAA, 0.1mM 2-mercaptoethanol and LIF.

To analyze fast TF dynamics, 5 nM PA-JF_549_ HaloTag ligand were added to cells for 20 min and then cells were washed with imaging medium for 3 times for 15 min inside the 37°C incubator. Stroboscopic single particle tracking was performed using a custom-built microscope equipped with an Olympus 60× NA 1.49 TIRF objective and a custom tube lense (LAO-300.0, Melles Griot), resulting in 100x overall magnification as described in ^125^. An iXon Ultra EMCCD camera (DU-897-CS0-BV, cooled to −80 °C, 17 MHz EM amplifiers, preamp setting 3, Gain 400, ROI-height: 35 pixels) was synchronized using a National Instruments DAQ board (NI-DAQ-USB-6363) at a frame time of 5 ms. 2 ms stroboscopic excitations of a 555 nm laser (CL555-1000-O with TTL modulation, CrystaLaser) was synchronized to the frame times of the camera via LabVIEW 2012 (National Instruments). The laser stroboscopically illuminated the sample using peak power densities of ~1.7 kW/cm^2^ using HiLO illumination of the nucleus ^126^. We adjusted the TIRF illuminator to the HiLO mode to deliver a highly inclined illumination to the cover glass with an incident angle smaller than the critical angle for TIRF. The resulting laser beam forms a thin laminated optical sheet (~3 μm in thickness) that causes lower out-of-focus excitation, and thus higher SNR compared to conventional Epi-illumination ^126^. PA-JF_549_ labels were photoactivated by 100μs-long excitation pulses of 407 nm light (50 W/cm^2^) every 50 ms. During the course of image acquisition, the pulse interval was shortened to 25 ms. 10,000 frames were typically recorded per cell. During imaging, cells were maintained at 37 °C and 5% CO_2_ using a Tokai-hit stage top incubator and objective heater.

We adopted a previously described analytical method—multiple target tracing (MTT) for SMT analysis ^127^. Briefly, single fluorescent emitters were detected using a stringent generalized likelihood ratio test with very low false discovery rate. Detected peaks were evaluated by a 2D Gaussian fit with the enabled deflation loops. Localized particles were connected to generate trajectories by thresholding the diffusion coefficient by taking past history into account under maximum likelihood test. Blinking and photobleaching were also considered during tracking process. The single molecule tracking parameters are described previously ^104^. The jump length histogram analysis and kinetic model fitting was described previously ^128^.

### Statistical Analysis

Unless stated specifically, data are presented as Mean ± SEM (standard error of the mean) with statistical significance (* p < 0.05, ** p < 0.01, *** p <0.001). We used non-parametric two-tailed Mann-Whitney *U* tests unless indicated in the figure legends. For cumulative distribution function, we also used the two-sample Kolmogorov-Smirnov test.

### Polymer Modeling

We introduced an off-lattice polymer model to study chromatin structure and dynamics. Chromatin was modeled as beads on a string, with each bead corresponding to a 5kb long genomic loci. A total of 600 beads was used to represent a 3Mb long segment. We further partitioned the chromatin into three regions of equal length, with the middle one as an ATAC-poor segment, and the two at the ends as ATAC-rich segments. ATAC-rich segments correspond to open chromatin and contain a total of 12 CTCF-binding sites that are uniformly distributed along the chromatin with a separation of 15 beads. CTCF sites start at the 15^th^ and 415^th^ bead for the two segments, respectively. For simplicity, we assumed that the ATAC-poor segment does not contain open chromatin and is therefore free of CTCF sites.

The effect of Cohesin on 3D chromatin organization was modeled using covalent bonds between two chromatin segments bound by a Cohesin molecule. Positions of Cohesin molecules on the chromatin were determined using the loop extrusion model following the same simulation protocol outlined by Mirny and coworkers ^13^(see below for details).

Encouraged by our experimental observations of the double bromodomain and LCD-containing scaffold protein BRD2 in ACD organization, we explicitly simulated 400 scaffold protein molecules containing both DNA binding domain and LCD to account for the role of scaffold-protein mediated protein-DNA and protein-protein interactions in organizing the chromatin. Each protein was modeled as a tetramer to mimic the conformational flexibility and disorder of LCDs. These proteins preferentially bind with open chromatin (like BRD2) and exhibit a weak self-interaction to promote aggregation and clustering. They can exist either in an “active” or “inactive” state. Active protein molecules contain binding domains that can form specific interactions with chromatin. Inactive proteins that do not form specific interactions with chromatin serve mainly as crowding molecules that mimics the crowded environment of the nucleus. They also ensure that the impact of crowding on chromatin volume is similar in different setups when the concentration of active proteins is varied upon Cohesin removal (see *Section:* Detailed setup of simulation systems for details).

In the following, we provide details on the model energy function, dynamical simulations, and the setup for mimicking the wild type system and various perturbations considered in our experiments.

### Details on Model Energy Function.

The simulation system considered here consists of chromatin, Cohesin molecules, CTCF binding sites and scaffold proteins. Its potential energy function includes chromatin self-interaction, Cohesin mediated chromatin interaction, protein-protein, and protein-chromatin interaction, and is defined as

$$U_{\text{model}}=U_{\text{chromatin}}+U_{\text{Cohesin}}+U_{\text{protein}}+U_{\text{protein}-\text{chromatin}}.$$

In the following we provide mathematical expressions for each term using reduced units with length scale *σ* = 30 nm and energy scale *ϵ* = *k_B_T*.

#### Chromatin model and self-interaction.

The potential energy function for the chromatin includes four terms,

$$U_{\text{chromatin}}=U_{c-\text{bond}}+U_{\text{angle}}+U_{\text{ex}}+U_{\text{inactive}}.$$

*U*_c–bond_ (*r*) is the bonding potential between neighboring beads to ensure the connectivity and is defined as

(Equation S1) $$U_{c-\text{bond}}(r)=K_{2}(r-r_{0}{)}^{2}+K_{3}(r-r_{0}{)}^{3}+K_{4}(r-r_{0}{)}^{4}$$

where $K_{2}=K_{3}=K_{4}=20\;\frac{\epsilon }{\sigma^{2}}$ and *r*_0_ = 2.0*σ*.

*U*_angle_ (*θ*) is the angular potential between every three consecutive beads to enforce the persistent length of the polymer. It adopts the following form

(Equation S2) $$U_{\text{angle}}(\theta )=K_{a}(1-\cos(\theta -\pi ))$$

where *K*_a_ = 2*ϵ*.

*U*_ex_ is the non-bonded potential to describe the excluded volume effect. It is applied between pairs of beads from ATAC-rich segments, and is modeled with the Weeks-Chandler-Andersen (WCA) potential

(Equation S3) Uex(r)={4ϵ[(σr)12−(σr)6]+ϵ,r≤21∕6σ0,r>21∕6σ}

*U*_inactive_ is the non-bonded potential between beads from the ATAC-poor segment. It enforces a condensed conformation for the segment and adopts the form of a truncated and shifted Lennard-Jones potential

(Equation S4) Uinactive(r)={4ELJ[(σr)12−(σr)6]+Ecut,r≤rcut0,r>rcut}

where *E*_LJ_ = 0.62*ϵ* and *r*_cut_ = 2.5*σ*. $E_{\text{cut}}=-4E_{\text{LJ}}\left[{\left(\frac{\sigma }{r_{\text{cut}}}\right)}^{12}-{\left(\frac{\sigma }{r_{\text{cut}}}\right)}^{6}\right]$ and ensures energy continuation at the distance *r*_cut_.

#### Cohesin mediated chromatin interaction.

Cohesin was modeled as a dimer and each monomer can bind with a separate chromatin bead. For each pair of chromatin beads occupied by the same Cohesin molecule, a harmonic bond of the following form is applied between them

(Equation S5) $$U_{\text{Cohesin}}(r)=K_{b}(r-r_{0}{)}^{2}$$

where *K_b_* = 4*ϵ* and *r*_0_ = *σ*.

#### Protein model and self-interaction.

We modeled scaffold molecules as tetramers with the two end monomers as binding domains. Binding domains interact favorably with specific protein binding sites on the chromatin, while non-binding domains show no such preference. In addition, a favorable interaction is introduced between binding domain themselves to promote protein-protein interaction. The energy function for protein is defined as

(Equation S6) $$U_{\text{protein}}=U_{p-\text{bond}}+U_{\text{angle}}+U_{\text{ex}}+U_{p-p}$$

The bonding potential between neighboring protein monomers is defined as

(Equation S7) $$U_{p-\text{bond}}(r)=-0.5KR_{0}^{2}\;\text{ln}\left[1-{\left(\frac{r}{R_{0}}\right)}^{2}\right]+U_{\text{WCA}}(r)$$

where $K=30\;\frac{\epsilon }{\sigma^{2}}$ and *R*_0_ = 1.5*σ*. *U*_WCA_ is the same Weeks-Chandler-Andersen potential as that defined in Equation S3.

The angular potential *U*_angle_ (*θ*) is the same as that for chromatin (Equation S2). We used the same non-bonded potential *U*_ex_ defined in Equation S3 for excluded volume effect between pairs of non-binding beads and between non-binding and binding beads.

The final term *U*_p–p_ describes the favorable protein-protein interaction between binding domains and is modeled using the same truncated and shifted Lennard-Jones potential defined in Equation S4, except that the energy scale *E*_LJ_ = 2.5*ϵ*. We tested different values for the strength of protein-protein interactions, and found that under current model settings, *E*_LJ_ = 2*ϵ* is too weak to induce any chromatin clustering. On the other hand, *E*_LJ_ = 3*ϵ* is too strong and proteins will form clusters without the presence of chromatin. *E*_LJ_ = 2.5*ϵ* captures the effect that chromatin may act as nucleation sites for protein clustering.

#### Protein-Chromatin Interaction.

The last term in the Hamiltonian, *U*_protein–chromatin_ describes the protein-chromatin interaction. The binding sites for scaffold proteins are located in the middle of two neighboring CTCF-binding sites, and a total of 15 sites are included in each ATAC-rich segment. For the specific interaction between protein binding domains and their binding sites on chromatin, a truncated and shifted Lennard-Jones potential defined in Equation S4 with *E*_LJ_ = 3*ϵ* is used. Otherwise, *U*_ex_ defined in Equation S3 is applied to model non-specific interactions between protein molecules and chromatin.

### Details on Dynamical Simulations.

Following Mirny and coworkers ^13^, we used a two-step algorithm to perform dynamical simulations and to consider the impact of Cohesin molecules on chromatin organization via loop extrusion. First, we carried out one-dimensional (1D) stochastic simulations of loop extrusion to collect a set of Cohesin positions along the chromatin. Next, for each set of Cohesin configurations, we performed equilibrium molecular dynamics simulations to sample three-dimensional (3D) conformation of chromatin.

#### 1D simulation of Cohesin extrusion.

To determine the genomic locations of Cohesin molecules along the chromatin, we performed stochastic simulations of the extrusion model ^13,14^ using the Gillespie algorithm ^129^. The set of chemical reactions in this 1D model includes Cohesin binding, unbinding and extrusion ^13,130^. Cohesin molecules are modelled as two linked subunits, each of which occupies a lattice site. Cohesin molecules can bind onto the chromatin at a single lattice site or two adjacent sites with a rate of *k*_on_, provided that neither site is occupied by another Cohesin or CTCF molecule. The two binding modes ensures the formation of both odd and even sized loops. A constant rate *k*_on_ is used with the assumption that the nuclear protein concentration is high and remains constant. Once bound, Cohesin molecules can extrude to neighboring empty sites by moving both subunits in opposite directions at a rate of *k*_extr_, provided that the target sites are not occupied by other Cohesin subunits or CTCF. If the target sites are occupied by a CTCF molecule, the Cohesin is blocked but can move pass the CTCF molecule with a probability of 0.01, since these barriers are permeable because of protein unbinding. Bound Cohesin molecules can also dissociate from the chromatin at a rate of *k*_off_. Cohesin molecules were also assumed to fall off from the chromatin once they reach the two end beads.

To avoid boundary effects of the two end CTCF sites in each of the two ATAC-rich segments, we assumed that the first CTCF site does not block extrusion from the left side, and the last CTCF site does not block extrusion from the right side.

Numerical values for the rates used in the simulations are *k*_extr_ = *τ*^−1^, *k*_off_ = 0.04*τ*^−1^ and *k*_on_ = 0.002*τ*^−1^ respectively. From these rates, Cohesin processivity is estimated to be 250kb, and the mean separation between two neighboring Cohesin molecules is 90kb. These values are comparable to the optimal ones reported by Mirny and coworkers that were obtained by fine tuning the agreement between simulated and Hi-C contact maps ^13^. Since extrusion rate has been estimated to be ~600bp/s ^131^, the simulation time unit *τ* can be converted to real unit $\frac{5kb}{600bp∕s}\approx 8.3s$.

Two independent simulations were performed for the wild-type and CTCF depletion system, respectively. For each simulation, we recorded Cohesin positions along the genome at every time interval *τ*. The first 4-million-*τ* long segments of these trajectories were discarded as equilibration. Following the equilibration period, 100 sets of Cohesin positions were collected.

#### 3D simulation of chromatin organization.

We carried out constant temperature and constant volume (NVT) simulations to model chromatin organization in 3D space using the software package LAMMPS ^132^. These simulations use the molecular dynamics algorithm to sample the equilibrium distribution of the potential energy function *U*_model_ defined in the *Section:* Details on Model Energy Function. Temperature for all simulations were maintained at a constant *T* = 1.0 through Langevin dynamics with a damping coefficient of 0.5*τ*_B_, where *τ*_B_ is the time unit. The time step is set to be *dt* = 0.01*τ*_B_. All simulations were carried out in a cubic box of length 60*σ* with periodic boundary conditions. Initial configurations of the chromatin polymer for all simulations are generated with a 3D random walk. Initial coordinates for protein molecules in the solution are also generated randomly.

The timescale in polymer models can be mapped onto physical units by matching the simulated diffusion constant with that in the nucleus. Specifically, the diffusion constant can be estimated using the fluctuation dissipation theorem as $D=\frac{k_{B}T}{\zeta }$. In Langevin dynamics, the friction coefficient $\zeta =\frac{m}{\xi }$, where *ξ* = 0.5*τ_B_* is the damping coefficient. The simulated diffusion constant therefore is $D=0.5\frac{\sigma^{2}}{\tau_{B}}$, or $\tau_{B}=0.5\frac{\sigma^{2}}{D}$. In the meantime, we can estimate the diffusion constant using the Stokes equation $D=\frac{k_{B}T}{\zeta }=\frac{k_{B}T}{3\pi \eta \sigma }$, where *η* is the viscosity of the nucleoplasm, and has been reported to be on the order of 10 cP. Therefore, the time unit can be estimated as $\tau_{B}=0.5\frac{\sigma^{2}}{D}=\frac{3\pi \eta \sigma^{3}}{2k_{B}T}=0.3\text{ms}$.

In extrusion-only, wild-type and CTCF-depletion systems, for each one of the 100 sets of Cohesin configurations obtained from the 1D simulations, we carried out a simulation that is 4 × 10^5^*τ*_B_ in length. For the Cohesin-depletion system, a total of eight independent 10^6^*τ_B_*-long simulations were performed. Configurations along the simulation trajectory were stored at every 2000 time-steps for analysis.

### Detailed setup of simulation systems

#### Extrusion-only model.

In accord with the original extrusion model, we did not consider the effect of scaffold proteins in 3D simulations. Therefore, all scaffold molecules are present in the inactive state.

#### Wild type.

Here, we modeled 75 protein molecules as “active” ones that contain binding domains to form specific interactions with chromatin. The concentration of active protein molecules is therefore 20.4 nmol/L, which is in the range of the reported nuclear protein concentration ^133^.

#### CTCF depletion.

In this system, CTCF sites were removed from the two ATAC-rich segments during 1D extrusion simulations. CTCF depletion will affect the average loop size formed by Cohesin molecules, which in turn affects the chromatin organization in 3D. For 3D simulations, we kept the setup for scaffold molecules and their binding sites unchanged as those in the wild-type system. This is motivated by the observation that CTCF depletion has negligible effect on chromatin accessibility in the enhancers and promoters as described previously ^24^.

#### Cohesin depletion:

For this setup, no 1D simulations were performed since loops cannot form without Cohesin molecules.

For 3D simulations, we increased the number of active protein molecules from 75 to 200 that corresponds to an increase of the concentration from 21.3 to 56.9 nM. This increase of protein concentration is mainly motivated by the observed elevation of local BRD2 concentration binding to chromatin upon Cohesin depletion (Fig.3d).

### Robustness of simulation parameters

A significant finding of the current study is that the extrusion model is insufficient to explain the enhanced clustering of accessible chromatin upon Cohesin removal. The rational for this is that loops enforced by Cohesin molecules tend to promote inter-chromatin contacts and favor collapsed chromatin conformations. Removing Cohesin in the extrusion model will always decrease chromatin compaction and, therefore, clustering. This conclusion should be general and independent from specifics of the model, as we show below.

#### Cohesin concentration:

In order to demonstrate the robustness of this conclusion with respect to Cohesin concentration, we varied the binding rate *k*_on_ in 1D simulations from 0.002*τ*^−1^ to 0.004*τ*^−1^ and 0.001*τ*^−1^. These setups lead to normal, dense and light densities that correspond to a mean separation of 90kb, 180kb and 45kb between Cohesin molecules. As shown in Extended Data Fig.3i, for all the parameters explored, Cohesin removal always leads to a decreased clustering for chromatin.

#### Locations and density of CTCF-binding sites:

In order to demonstrate the robustness of this conclusion with respect to CTCF locations, we performed additional simulations with an alternative distribution of the CTCF sites in each of the two active (ATAC-rich) segments. We kept the number of CTCF-binding sites the same but randomly generated an alternative set of CTCF sites on each of the active domains. In addition, we also performed simulations in which the total number of CTCF-binding sites on the chromatin was varied from 24 to 12 or 36. As shown in Extended Data Fig.3h, in all cases, removing Cohesin leads to a decreased clustering of accessible chromatin.

We further evaluated the robustness of simulation results from the polymer model that combines loop extrusion with the effect of scaffold protein molecules as follows.

#### CTCF positions:

To test the robustness of results relative to CTCF locations, we performed additional simulations using the randomly generated CTCF sites mentioned above. As shown in Extended Data Fig.12d-g, the trends in chromatin clustering, 3D distance between active domains and volume of individual domains are preserved and qualitatively similar to those shown in Fig.5b-d.

#### Protein concentrations:

One assumption of the polymer model is the increase of the concentration for scaffold proteins upon Cohesin removal. To investigate the robustness of simulation results with respect to this concentration change, we tested a series of scaffold molecule concentrations and compared all the metrics that we used: pair auto-correlation function, simulated FISH measurements and volume distributions of single ATAC-rich segments. We found that beyond a certain threshold (56.9 nM, i.e., an increase by a factor of 2.7), all results are stable regardless of the protein concentration (Extended Data Fig.12a-c).

### Structural analysis of chromatin

#### Contact Probability map:

We calculated the contact probability maps to highlight the contact difference between different perturbation conditions. We calculated the contact probability *P*(*r*) of each pair of beads with separation of *r* using the following expression ^134^:

P(r)={12[1+tanh(σ(rc−r))],ifr≤rc0,forr>rc}

Where *r_c_* = 1.76 and *σ* = 3.72.

#### Pair auto-correlation function *g*(*r*):

*g*(*r*) was calculated as the probability distribution of pair-wise distances using the standard algorithm ^135^. We further normalize it with the corresponding distribution of hard spheres to highlight the clustering. The hard sphere *g*(*r*) was calculated from a uniform distribution for the same number of beads in a cubic box of the same size as the simulation box.

#### Volume for active domain:

To calculate the volume of a given chromatin conformation, we determined the convex hull for all the 3D points that correspond to the Cartesian coordinates of chromatin monomers. The volume of the chromatin domain is then set as the volume of the convex hull.

## Extended Data

## Supplementary Material

Table S1 **Supplemental Table 1.** Table of genes with significant change of expression after 6-hour acute depletion of BRD2.

Table S3 **Supplemental Table 3.** Table of summarized next-generation sequencing datasets in this study.

Table S2 **Supplemental Table 2.** Table of genes with significant change of expression after 6-hour acute depletion of BRD4.

Movie S1 **Supplemental Movie 1.** 3D representation of 3D ATAC PALM localizations in untreated control (left) or RAD21 (right) depleted mESCs. The color bar indicates localization density calculated by using a canopy radius of 250 nm.

Movie S2 **Supplemental Movie 2.** 3D representation of SOX2 stable binding sites localizations in live mESCs for untreated control (left) or RAD21 depletion (right) by the lattice light-sheet microscope. HaloTag labeled SOX2 was stably expressed in Rad21-mAID-eGFP cells and detected by low concentration of JF_549_ HaloTag ligand. The stable SOX2 binding events with long residence time (>4s) were reconstructed in 3D, with the localization density color coded.
